# The Combination of Scutellaria baicalensis Georgi and *Sophora* japonica L. ameliorate Renal Function by Regulating Gut Microbiota in Spontaneously Hypertensive Rats

**DOI:** 10.3389/fphar.2020.575294

**Published:** 2021-02-12

**Authors:** Yiqing Guan, Kai Chen, Dongling Quan, Liangqi Kang, Danni Yang, Huanxian Wu, Mengqiu Yan, Shaoyu Wu, Lin Lv, Guohua Zhang

**Affiliations:** ^1^ School of Traditional Chinese Medicine, Southern Medical University, Guangzhou, China; ^2^ School of Pharmaceutical Sciences, Southern Medical University, Guangzhou, China

**Keywords:** Scutellaria baicalensis Georgi, *Sophora* japonica L, hypertensive nephropathy, gut microbiota, traditional Chinese medicine

## Abstract

Chronic kidney disease (CKD) is becoming a notable health concern globally. The combination of Scutellaria baicalensis Georgi (SB) and *Sophora* japonica L. (SJ) has been demonstrated to have anti-hypertensive effects and improve kidney injury clinically. This study aimed to explore the renal protective effect of the combination of SB and SJ against CKD and clarify the potential mechanisms. Male spontaneously hypertensive rats (SHR) were used to induce hypertensive nephropathy and were treated with SB or SJ separately or in combination for 15 weeks, and an antibiotic group was used for a rescue experiment. Blood pressure, serum or urine biochemical markers, serum inflammation factors, short-chain fatty acids (SCFAs), indoxyl sulfate (IS), and oxidative stress indicators were assessed. Western blot analysis was performed to determine the expression of intestinal tight junction proteins, including occludin and ZO-1. The mRNA expression of the SCFAs receptors olfactory 78 (Olfr78) and G protein-coupled receptor 41 (GPR41) was determined by quantitative real-time PCR. Gut microbiota profiles were established via high-throughput sequencing of the V3-V4 region of the bacterial 16S rRNA gene. SB and SJ significantly ameliorated the severity of renal injury induced by hypertension. The combination also decreased the ratio of Firmicutes/Bacteroidetes, increased the relative abundance of *Lactobacillus*, and reduced that of Clostridiaceae. The intestinal barrier was improved, and the change in dominant bacteria reduced IS accumulation and further inhibited oxidative stress activation in kidneys. SB and SJ increased SCFAs production, inhibited inflammatory factor release, and regulated blood pressure by decreasing the expression of Olfr78 and increasing that of GPR41, then alleviated kidney damage. This research demonstrated the positive effects of SB and SJ in a rat model of hypertensive nephropathy, indicated that the treatment of SB and SJ by improving the intestinal barrier function, increasing SCFAs, reducing inflammation, decreasing IS, and inhibiting oxidative stress reactions.

## Introduction

With the increasing incidence of chronic kidney disease (CKD), this condition has developed into a major global public health problem. According to the latest Global Nephropathy Health Report released by the World Renal Congress in 2017, one in ten people worldwide develop kidney disease (Bello et al., 2017). If hypertension and kidney damage exist simultaneously, they will exacerbate one another, thus aggravating the resulting damage. In regards to remedies for progressive CKD, the application of traditional Chinese medicine (TCM) offers possible treatments, and the foundational and clinical research in this field have been shown as effective in cardiovascular treatment (Hao et al., 2017).

In recent years, accumulating evidence has shown intestinal flora imbalance and impaired intestinal barrier function in CKD patients (Vaziri et al., 2013), It has been proposed that there is a close relationship between the intestine and the kidney, which is called “intestinal-renal axis” (Yang et al., 2018). The metabolites of intestinal flora are major mediators connecting the “intestinal-renal axis”. In addition, more and more evidence has claimed that CKD is considered to be caused by immune cells, which are activated by the intestinal flora to cause low-grade inflammation and influence the brain, cardiovascular system, and renal system through circulation (Akchurin and Kaskel, 2015; Santisteban et al., 2015; Shankland and Jefferson, 2017). Accordingly, improving gut dysbiosis would be a potential method for the prevention and treatment of this chronic illness.

Previous studies have confirmed that many traditional Chinese medicinal formulas have protective effects on hypertensive kidneys. However, the inclusion of numerous herbs in formulas is detrimental to controlling medicinal quality and exploring specific mechanisms, so it’s necessary to simplify the herbal formulas in accordance with the goal of modernizing TCM. Thus, guided by the TCM theory and based on multitudinous clinical experiences, a new combination of the treatment for hypertensive nephropathy was selected comprising of two individual herbs: Scutellaria baicalensis Georgi (SB) and *Sophora* japonica L. (SJ). SB, one of the most widely used traditional Chinese herbal medicines, has been used to treat inflammation (Ma et al., 2013), suppress fibrosis, lipid peroxidation (Kong et al., 2011; Pan et al., 2012) and closely related to intestinal flora. SJ has been historically used over 3,000 years to treat many diseases, including hypertension, and modern pharmacological studies have shown that the active components and/or crude extracts of SJ exhibit a wide range of pharmacological actions, such as cardiovascular effects as well as anti-inflammatory, antioxidant, antibacterial, antiviral, hemostatic, and anti-atherosclerotic effects (Ishida et al., 1989; Byung et al., 1996). In China, SB and SJ are used for treating hypertension and kidney injury, which can ameliorate the symptoms of hematuria and proteinuria. However, its underlying mechanism is still unknown.

In this work, an animal model of spontaneously hypertensive rats (SHR) was used to evaluate the therapeutic effects of SB and SJ on hypertensive kidney damage progression and the intestinal flora environment. Gut microbiota analysis was employed to further explore the involvement of the gut microenvironment in protecting the kidneys.

## Methods and Materials

### UPLC-MS

Scutellaria baicalensis Georgi (batch number: 8045841) and *Sophora* japonica L. (batch number: 7110901) produced by EFONG Pharmaceutical Company, Ltd. (Guangdong, China) were purchased from Nanfang Hospital, Southern Medical University, and identified by Chuanmin Liu, a professor in School of Traditional Chinese Medicine in Southern Medical University. First, 0.3 g sample was placed in a 10 ml Eppendorf tube, dissolved by adding 8 ml of 50% methanol-water solution, and sonicated at 25°C for 30 min. After resting the sample for 5 min, 1 ml of the supernatant was placed in a 2 ml tube, centrifuged at 13,000 rpm for 10 min, passed through a 0.22 μm microporous membrane, and loaded into a 1.5 ml auto-injection bottle to obtain the sample extract. Blank control samples were obtained under the same conditions. Samples were refrigerated at 4°C and removed before analysis (storage time must not exceed 24 h). For chromatographic analysis, the Shimadzu LC-30A was used with a C18 column (1.8 μm, 2.1 × 100 mm) at 40°C with a flow rate of 0.3 ml/min and an injection volume of 5 μL. The mobile phase used the gradient elution method with acetonitrile 0.1% formic acid aqueous solution. A gradient elution program was utilized for the separation and determination.

Mass spectrometry was performed on the AB Sciex Triple TOF 5600+ in the positive electrospray ionization mode with the ion source setting at 5500 V and 600°C. The declustering voltage, collision energy (CE), and collision energy expansion (CES) were 100 V, 35, and 15 eV, respectively. Nitrogen was used as the atomizing gas, and auxiliary gas 1, auxiliary gas 2, and the air curtain gas were set at 60, 50, and 40 PSI, respectively. The primary ion mass spectrometer scan range was 50–1,000. The information dependent acquisition (IDA) set the six highest peaks with a response value exceeding 100 cps to perform the secondary mass spectrometry scan. The product ion scan range was 50–1,000, and dynamic background subtraction (DBS) was enabled.

For the ionization mode, the electrospray negative ionization mode was used with the ion source set at −4,500 V and 500°C The declustering voltage, CE, and CES were 100 V, 35, and 15 eV, respectively. Nitrogen was used as the atomizing gas, and auxiliary gas 1, auxiliary gas 2, and the air curtain gas were set at 60, 50, and 40 PSI, respectively. The primary ion mass spectrometer scan range was 50–1,000. IDA set the six highest peaks with a response value exceeding 100 cps to perform the secondary mass spectrometry scan. The product ion scan range was 50–1,000, and DBS was enabled.

### Animal and Administration

Twenty-week-old male SHR and Wistar-Kyoto (WKY) rats were used, the latter were negative control, each rat weighed 350 ± 20 g (the two strains share the same genetic background and blood pressure of WKY stabilizes at a low level). Both strains were purchased from VITAL RIVER Co., Ltd. (Beijing, China) and rats were housed under specific pathogen-free conditions with regular 12h diurnal cycles at 23 ± 2°C and 60 ± 5% relative humidity. All rats were fed with standard fodder according to GB14924.3-2010 and sterile water. The study protocol was reviewed and approved by the Animal Review Board of Southern Medical University, Guangzhou, China. The research was conducted in accordance with the Animal (Scientific Procedures) Act of 1986 and the institutional guidelines of the Southern Medical University for the care and use of animals. After one-week-acclimatization, 40 rats were randomly divided into eight groups as follows (n = 5 rats/group), and every five rats were in a cage.1) WKY, 2) SHR, 3) SHR + SB, 4) SHR + SJ, 5) SHR + SB + SJ, 6) SHR + captopril (CAP), 7) SHR + antibiotics (ABS), and 8) SHR + SB + SJ + antibiotics. SB and SJ were purchased from EFONG Pharmaceutical Company Ltd. (Guangdong, China). Captopril (Lot 1209031) was obtained from Bristol-Myers Squibb Company Ltd. (Shanghai, China). A single dose of SB (0.9 g/kg), SJ (0.9 g/kg), SB and SJ in combination (SB + SJ, 0.9 g/kg each), or captopril (13.5 mg/kg) was given to the groups separately once a day. The dosage of SB or SJ for rats was calculated according to the oral dosage of adults (the dosage of SB or SJ for adults is 10 g, respectively). To reduce the native microbiota load, and to determine the role of SB and SJ in reducing the imbalance of intestinal flora the antibiotics ampicillin (0.25 mg/ml), neomycin (0.25 mg/ml), metronidazole (0.25 mg/ml), and vancomycin (0.125 mg/ml) (Sigma, St. Louis, MO, USA) were dissolved in autoclaved water to feed rats. The treatment lasted for 15 weeks. Each week, we recorded body weight and monitored blood pressure (BP) via a tail-cuff plethysmograph (ALC-NIBP, Shanghai Alcott Biotech Co., Shanghai, China). At the end of the trial, we collected fecal samples from individual rats and stored them at −80°C promptly. Finally, the general condition of the rats was normal and all survived before sacrifice. Rats were fasted overnight and anesthetized with an intraperitoneal injection of urethane (1.0–1.2 g/kg), and sacrificed them. Then, the harvested plasma samples were centrifuged at 3,000 rpm for 15 min at 4°C and were stored at −80°C immediately until analysis. The intestinal and kidney tissues for histopathology analysis were quickly collected. These tissue sections were fixed with 4% paraformaldehyde for 48 h and then used for histopathological analysis.

### Biochemical Analysis

After 15 weeks, blood samples were collected from the tail vein of each rat, and the serum was centrifuged at 3,000 rpm for 15 min at 4°C. The supernatant was stored at −80°C for biochemical analysis. With metabolic cages, urine within 24 h was collected from rats. Biochemical kits for the detection of creatinine (Cr; product code: C011-2-1), blood urea nitrogen (BUN; C013-1-1), and urine microalbumin (mALB; E038-1-1) were purchased from Nanjing JianCheng Bioengineering Institute (Nanjing, China). Tumor necrosis factor alpha (TNF-α) for the detection of Cr, BUN, mALB, were purchased from Nanjing JianCheng. Interleukin 1β (IL-1β) ELISA kit (ml037361) was purchased from Shanghai Jichun Industrial Co., Ltd. (Shanghai, China). The assay kits for malondialdehyde (MDA; BB-4709) and catalase (CAT; BB-47044) were purchased from Guangzhou Yike Biological Technology Co., Ltd. (Guangzhou, China). Total superoxide dismutase (SOD; S0109) and glutathione peroxidase (GPx; S0056) were also purchased from Shanghai Biyuntian Biotechnology Co., Ltd. (Shanghai, China). The indoxyl sulfate (IS) detection kit (BS-E12258R1) was purchased from Jiangsu Boshen Biological Technology Co., Ltd. (Jiangsu, China). ELISA kits for acetic acid (BOS-dd02), propionic acid (BOS-46986), and butyric acid (BOS-46983) were purchased from Chengdu Yuannuo Tiancheng Technology Co., Ltd. (Chengdu, China).

### Histological Analysis

Kidneys, small intestines, and colons were fixed with 4% paraformaldehyde, embedded in paraffin, and sliced up, the thickness was 4 μm. These tissue sections were stained with hematoxylin and eosin (HE). Furthermore, kidney sections were stained with Masson’s trichrome and Periodic Acid Schiff. Colon sections were stained with Alcian blue. Immunohistochemistry (IHC) was used to analyze the relative expression of type I and type III collagen in kidneys and that of occludin in the colon. PBS instead of the first antibody was applied as negative control. The images were acquired by a lightmicroscopy (OLYMPUS, BX53, Japan). For immunofluorescence staining, the frozen colon sections which was selected to be our research target were incubated with anti-occludin (1: 100, Abcam, ab216327), the primary antibody, overnight at 4°C, then incubated with the appropriate secondary antibody, and counterstained with DAPI. A confocal microscope (FluoView1000; OLYMPUS) was used to capture the images.

### Western Blot Analysis

Western blotting was carried out using standard techniques. The intestinal and kidney tissues powder were homogenated in ice-cold loading buffer. and aliquots were stored at −80°C for Western blot analysis. The total protein were extracted using RIPA Lysis buffer (Fu De Biological Technology, Hangzhou, China). Supernatant were collected at 12,000 × g centrifugation for 30 min at 4°C. According to the manufacturer’s instructions, the BCA protein Assay Kit (Pierce Thermo-Scientific, Rockford, IL, United States) were used to determine the concentration of the protein. In Western blotting, we loaded 40–80 μg protein and the it was separated from the sample buffer using 8% SDS-PAGE, then transferred onto Polyvinylidene fluoride membranes (PVDF) (Millipore, Bedford, MA, United States). After blocking membranes for an hour with 5% bovine serum albumin (BSA) in Tris-buffered saline containing 0.1% Tween-20 at pH 7.6 (TBST), the membrane incubated with primary antibodies at 4°C overnight. Membranes were probed with the following primary antibodies: antibodies against collagen I (Abcam, Cambridge, United Kingdom; ab34710), collagen III (Abcam, ab7778), occludin (Abcam, ab216327), ZO-1 (Invitrogen, Carlsbad, CA, United States; 61-7300), GLI1 (Biogot, BS72669), and GAPDH (CST, Danvers, MA, United States; 2118S). After washing three times for 30 min in TBST, the membranes were incubated with the appropriate secondary antibody (1:4,000, product number: Anti-rabbit IgG, HRP-linked Antibody #7074 and Anti-mouse IgG, HRP-linked Antibody #7076, which were purchased from Cell Signaling Technology, Inc.) for 1 h at room temperature, and then washed three times for 30 min in TBST. The Images were scanned and visualized, blots were quantified by densitometric analysis using the software Image-Pro Plus 6.0, while normalizing the results by internal control with respect to GAPDH.

### Gut Microbiota Analysis

In order to extract DNA from fecal samples, all samples were immediately stored at -80°C, and the V3-V4 region of the bacterial 16S rRNA was sequenced in an Illumina MiSeq sequencer (300-bp paired-end reads). Quantitative Insights Into Microbial Ecology (Version 1.9.1) was used to analyze sequences. High-quality reads were selected, and all effective reads were clustered into operational taxonomic units (OTUs). The alpha diversity was analyzed by the rarefaction curve and chao1 index. The beta diversity was analyzed and visualized by principal coordinate analysis (PCoA) based on weighted UniFrac distance matrices. The linear discriminate analysis effect size (LEfSe) was performed to analyze differences of microbial diversity data in the samples. Phylogenetic investigation of communities by reconstruction of unobserved states (PICRUSt) analysis and categorization with the Clusters of Orthologous Groups of proteins (COG) and KEGG Orthology (KO) databases were used to analyze the predictive characterization of different microbial communities.

### Real-Time Reverse Transcription PCR

Total RNA was extracted from kidneys according to the protocol of the total RNA isolation kit (RNAqueous, AM1912, Invitrogen). The PrimeScript RT reagent kit was reversed into cDNA. Quantitative polymerase chain reaction (qRT-PCR) analysis was performed for Olfr78 and GPR41 genes. The primers were synthesized by Takara Bio Inc., and its sequences were listed in Supplementary Table S1. The PCR reactions were performed in a total volume of 20 μL containing 10 μL SYBR Premix Ex Taq II (Takara, Tokyo, Japan; Code No. RR820A), 0.8 μL forward primers, 0.8 μL reverse primers, 2 μL cDNA, and 6.4 μL RNase-free ddH2O. The mRNA expression levels were computed using the LC480 analyzer Real-Time PCR System. Each qRT-PCR reaction was performed in triplicate, the cycling conditions were: denaturation 95°C for 30 s followed by 30 cycles of 95°C for 5 s and extension at 60°C for 34 s. Subsequently, we used melting curves to monitor non-specific amplifications. GAPDH expression was used as an internal reference to normalize the results. The comparative threshold cycle (Ct) method used $2^{-\Delta \Delta \text{Ct}\;}$ method.

### Statistical Analysis

All data were presented as means ± standard deviation (SD). Multiple comparisons were performed using one-way ANOVA (*p* < 0.05) followed by Dunnett’s post-hoc *t*-test. All statistical analyses were performed using SPSS 21.0 software (SPSS Inc., Chicago, IL, United States).

## Results

### Chemical Composition of SB and SJ

**FIGURE 1:**
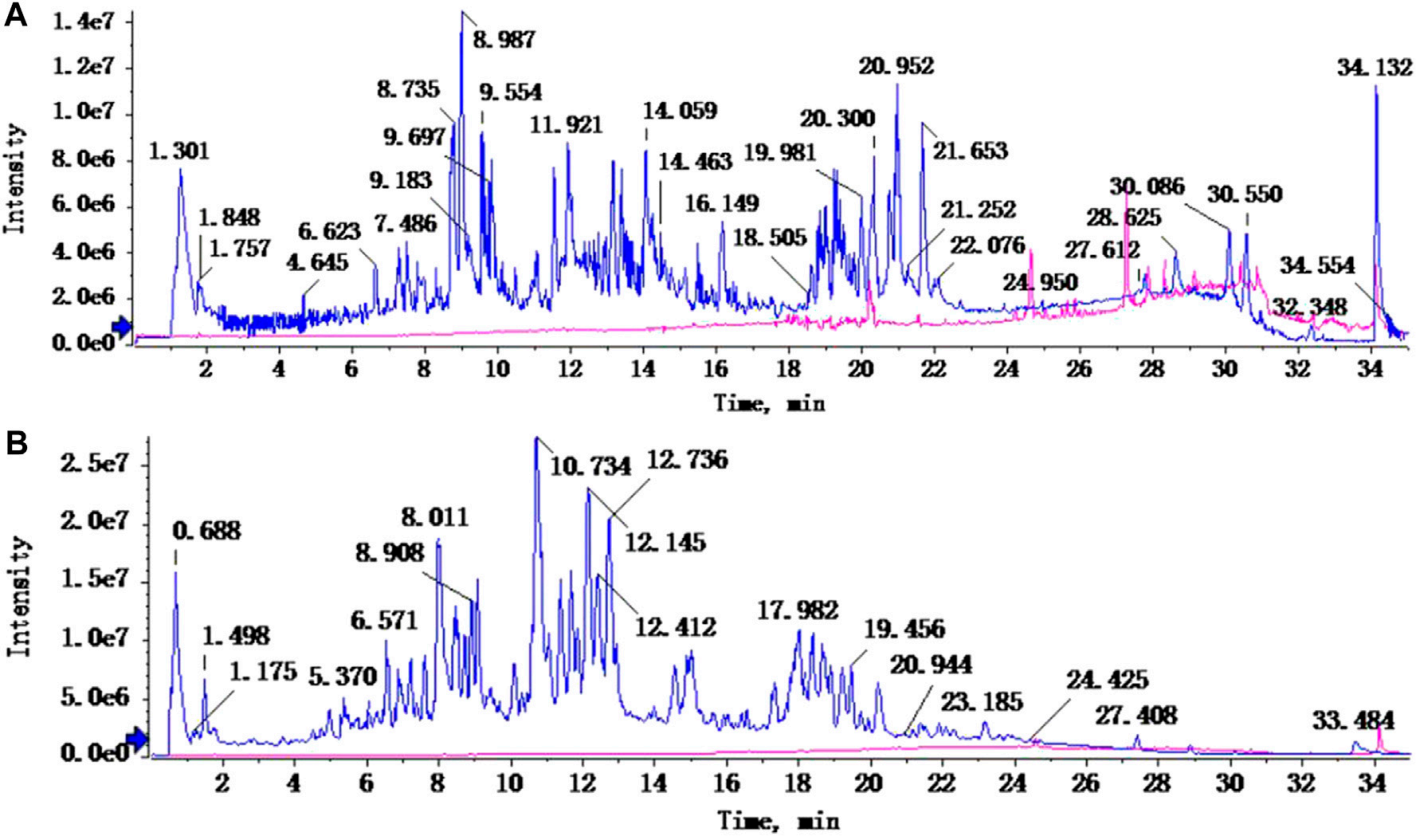
Total ion chromatogram of Scutellaria baicalensis Georgi and *Sophora* japonica L. under positive ion **(A)** and negative ion **(B)** modes.

In total, 31 components were detected in the extract in positive and negative ionization modes (Figure 1), which were ranked in order of the intensity of the mass spectrum response: rutin, wogonoside, kaempferol-3-o-rutinoside, baicalin, viscidulin I, quercetin, skullcapflavone II, dihydrobaicalein, dihydrobaicalin, baicalein, norwogonin, genistein, kaempferol, isorhamnetin, wogonin, viscidulin III, chrysin, soyasaponin Bb, sophora saponin III, 2′,5,6′,7-tetrahydroxyflavanonol, eriodictyol, 2,6,2′,4′-tetrahydroxy-6′-methoxychalcone, dianbaicalin, skullcapflavone, skullcapflavone I, viscidulin II, 7,2′6′-trihydroxy-5-methoxydihydroflavone, palmitic acid, dihydrooroxylin A, 2′,6′,5,7-tetrahydroxyflavan-one, and stearic acid. The concrete active ingredient test form of SB and SJ is shown in Table 1.

**TABLE 1:** The active ingredient test form of Scutellaria baicalensis Georgi and *Sophora* japonica L.

Number	Name	Formula	Chemical structural	Adduct	Found mass	Error	RT	Intensity
1	rutin	C27H30O16	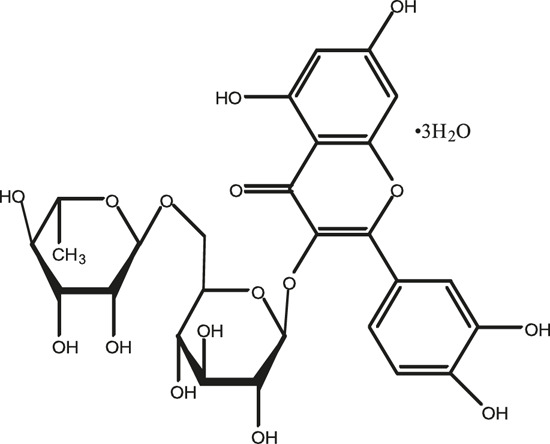	-H	609.14554	−0.9	8.02	5,385,074
2	wogonoside	C22H20O11	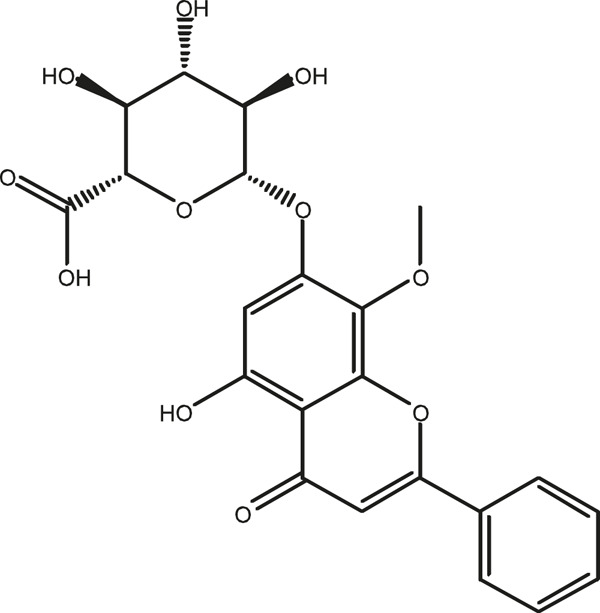	-H	459.0929	−0.8	12.73	3,402,314
3	kaempferol-3-o-rutinoside	C27H30O15	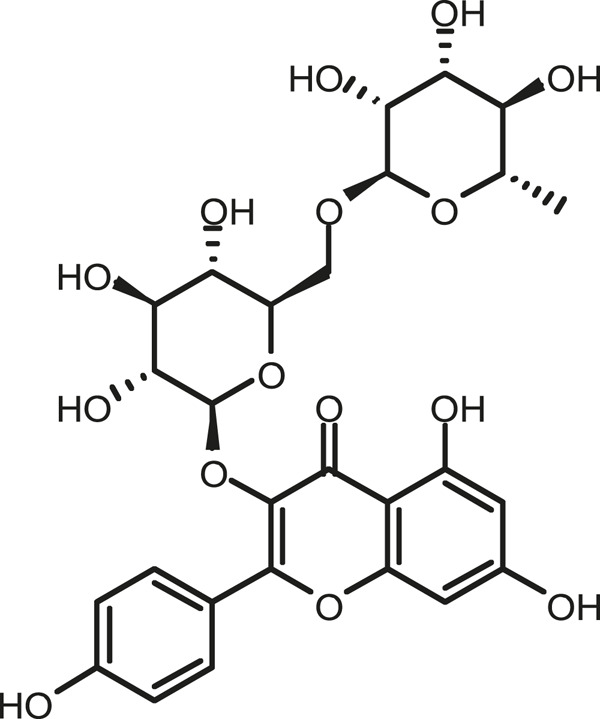	-H	593.15135	0.3	8.92	3,320,524
4	baicalin	C21H18O11	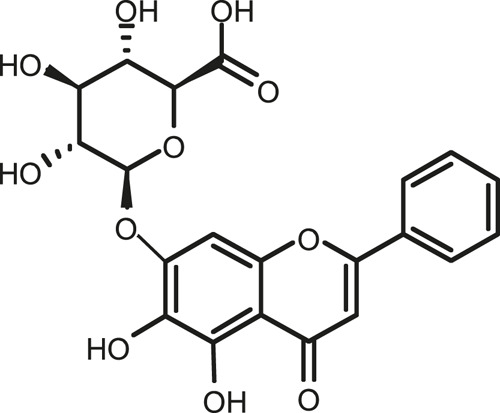	-H	445.07721	−1	10.74	3,002,573
5	viscidulin I	C15H10O7	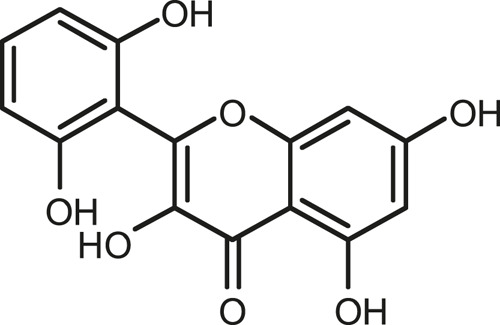	-H	301.03594	1.9	12.44	2,938,836
6	quercetin	C15H10O7	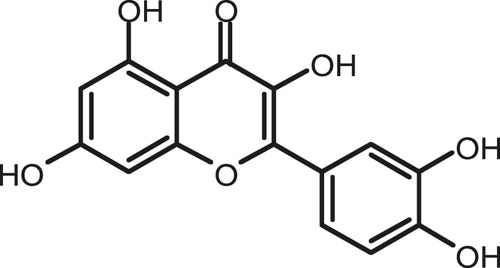	-H	301.03561	0.8	8.53	409,268
7	skullcapflavone Ⅱ	C19H18O8	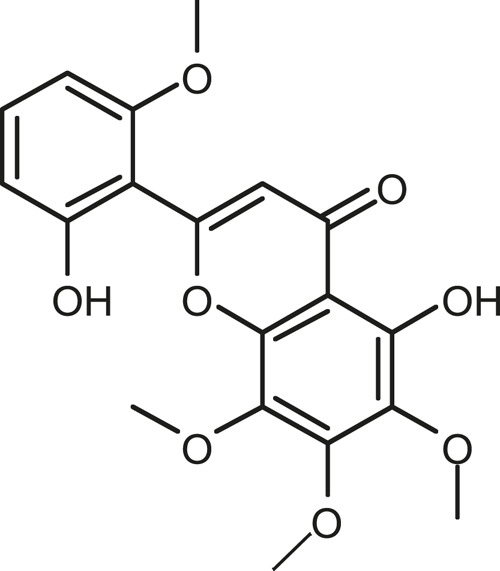	-H	373.09321	0.9	18.37	2,277,916
8	dihydrobaicalein	C15H12O5	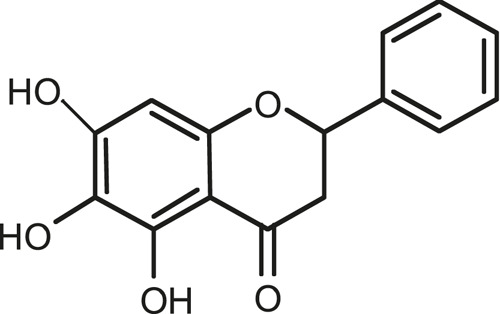	-H	447.09306	−0.5	11.39	2,203,825
9	dihydrobaicalin	C21H20O11	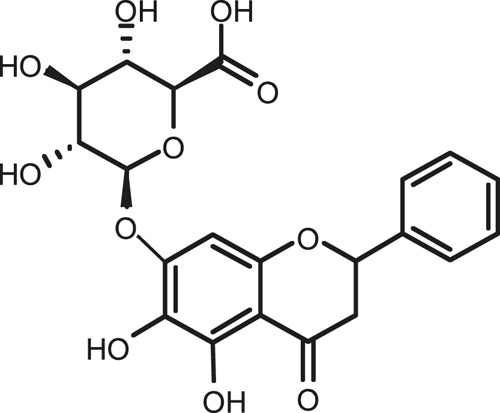	-H	447.09285	−1	9.34	486,989
10	baicalein	C15H10O5	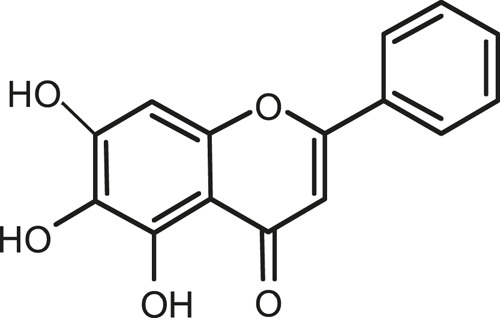	-H	269.04625	2.6	15.05	2,045,658
11	norwogonin	C15H10O5	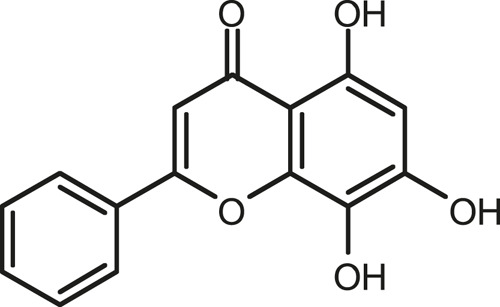	-H	269.04645	3.4	14.6	654,213
12	genistein	C15H10O5	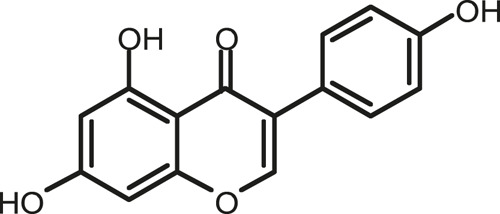	-H	269.04572	0.6	10.73	287,514
13	kaempferol	C15H10O6	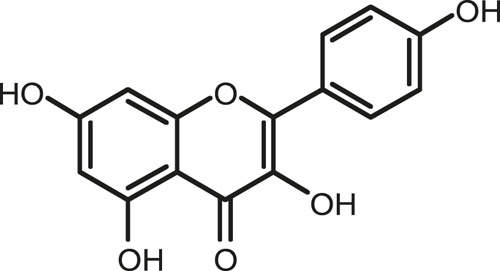	-H	285.04143	3.4	14.57	1,890,390
14	isorhamnetin	C16H12O7	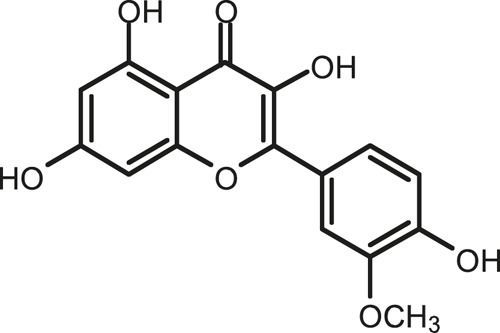	-H	315.05144	1.3	14.9	1,888,633
15	wogonin	C16H12O5	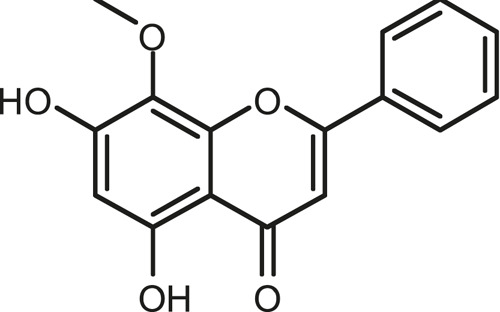	-H	283.06211	3.2	18.03	1,493,108
16	viscidulin III	C17H14O8	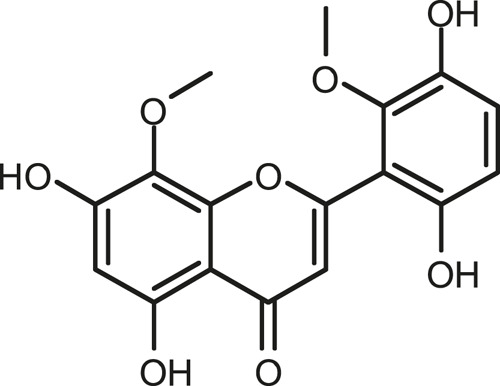	-H	345.0619	0.9	11.06	957,624
17	chrysin	C15H10O4	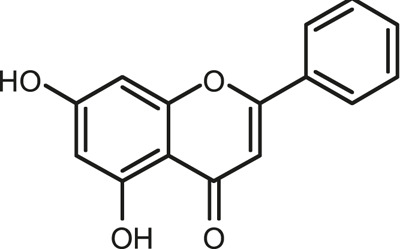	-H	253.05177	4.5	18.23	777,102
18	soyasaponin Bb	C48H78O18	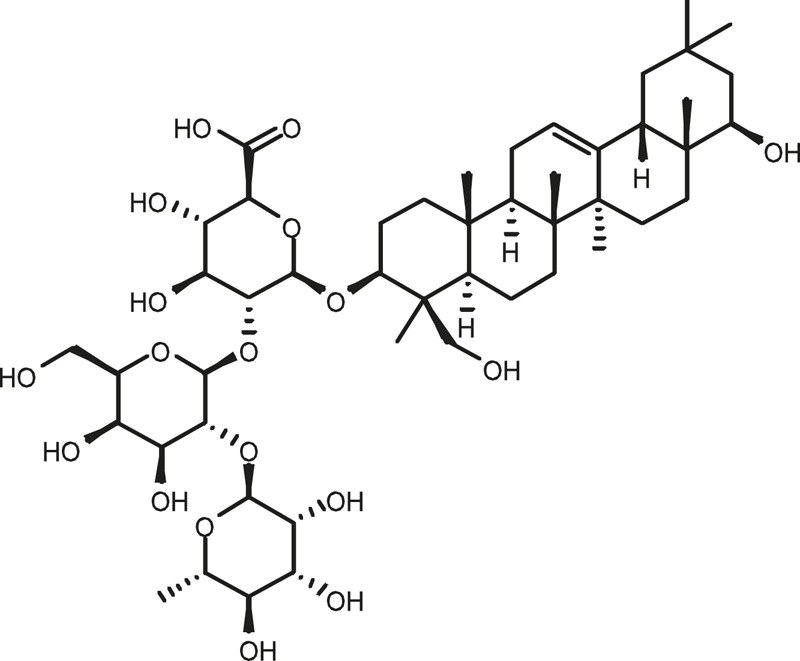	-H	941.51236	0.9	17.33	751,492
19	sophora saponin III	C48H78O17	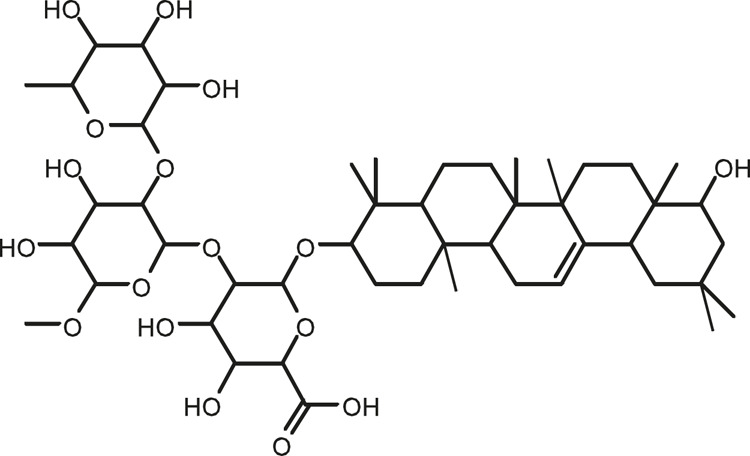	-H	925.51736	0.8	17.93	736,891
20	2′,5,6′,7-tetrahydroxyflavanonol	C15H12O6	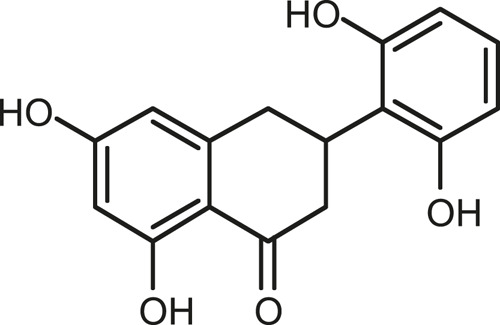	-H	287.05655	1.5	11.58	637,926
21	eriodictyol	C15H12O6	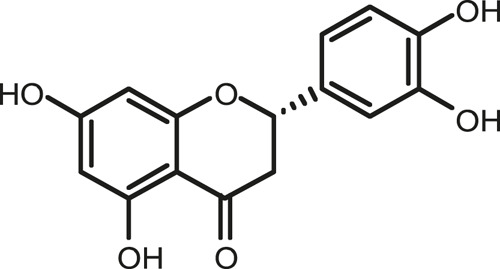	-H	287.05642	1.1	11.04	119,568
22	2,6,2′,4′-tetrahydroxy-6′-methoxychalcone	C16H14O6	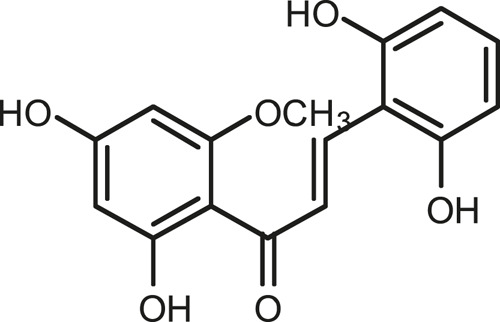	-H	301.07208	1.1	10.32	594,919
23	dianbaicalin	C16H14O6	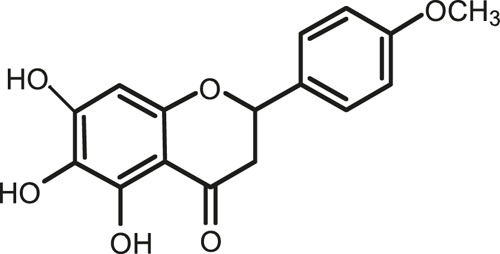	-H	301.07202	0.9	13.41	81,305
24	skullcapflavone	C18H16O7	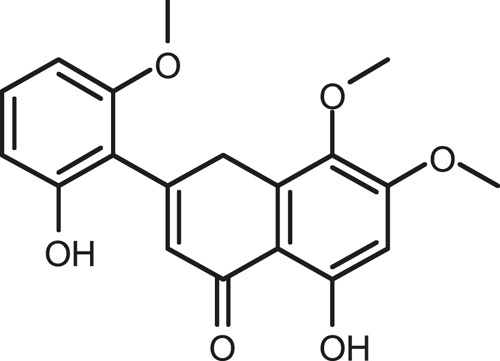	-H	343.08273	1.2	19.14	433,251
25	skullcapflavone I	C17H14O6	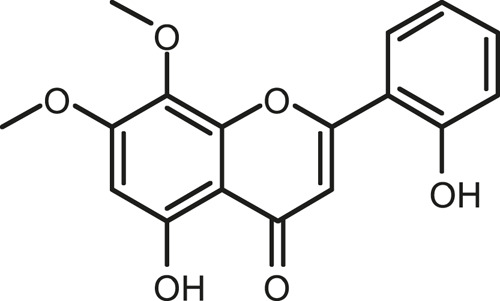	-H	313.07233	1.8	18.62	336,718
26	viscidulin II	C17H14O7	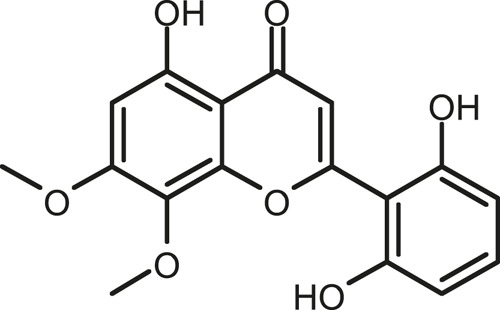	-H	329.06695	0.8	15.29	287,740
27	7,2′6′-trihydroxy-5-methoxydihydroflavone	C16H10O6	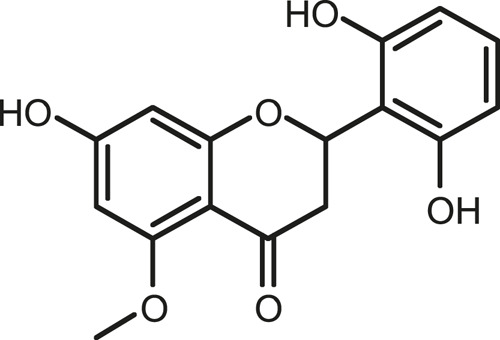	-H	297.04059	0.4	10.73	104,578
28	palmitic acid	C16H32O2	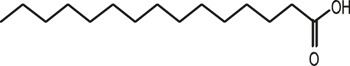	-H	255.23417	4.8	30.14	77,965
29	dihydrooroxylin A	C16H14O5	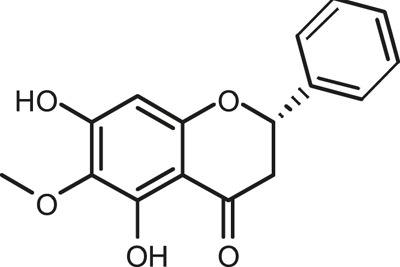	-H	285.07707	0.8	18.7	41,174
30	2′,6′,5,7-tetrahydroxyflavan-one	C15H14O5	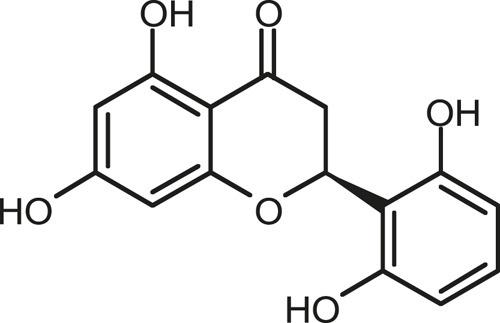	-H	273.07699	0.5	10.32	15,940
31	stearic acid	C18H36O2	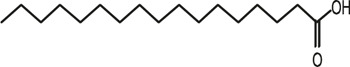	-H	283.26477	1.8	32.17	6,222

### The combination of SB and SJ can decrease the blood pressure in SHR.

The blood pressure of SHR group was significantly higher than that of WKY at the same age. As shown in Table 2, the SBP and DBP of SHR were higher than those in WKY rats (*p* < 0.001, respectively). After 15-week-treatment with medicine, the SBP and DBP of SHR in all treatment groups were significantly lower than that in SHR control group (*p* < 0.001, respectively). There was no significant difference in blood pressure between ABS group and SHR group, at the same time, the blood pressure in SB + SJ + ABS group was significantly decreased compared with that in SHR group. It was also pleased found that the antihypertensive effect of the SB + SJ group was better than that of the SB + SJ + ABS group. In the experiment, the antibiotics we used according to the reference can kill the most of intestinal flora in rats, the sequencing results of intestinal microflora also showed that there were still intestinal microflora in rats after treating with antibiotics. The antihypertensive effect of SB + SJ + ABS was significantly different from that of SB + SJ (*p* < 0.01), that meant the antihypertensive effect of SB + SJ was influenced after the intervention of intestinal flora, which indicated that the antihypertensive effect of SB + SJ may be accomplished through the flora.

**TABLE 2:** Effects of thecombination of SB and SJ on body weight and blood pressure of SHR.

Group	WKY	SHR	SB	SJ	SB + SJ	CAP	ABS	SB + J + ABS
Body weight(g)	352.9 ± 9.2	317.2 ± 9.0	329.0 ± 15.1	330.6 ± 12.3	326.9 ± 10.3	336.3 ± 13.9	332.2 ± 25.1	333.8 ± 16.3
SBP (mmHg)	120.54 ± 3.42	189.35 ± 2.68^###^	165.03 ± 3.04^***^	163.75 ± 3.76^***^	153.91 ± 2.35^***^	137.81 ± 2.04^***^	190.78 ± 3.16	162.12 ± 3.00^*** &&^
DBP (mmHg)	103.77 ± 1.38	144.06 ± 2.08^###^	133.38 ± 0.78^***^	130.40 ± 0.77^***^	124.27 ± 1.25^***^	114.46 ± 0.98^***^	146.24 ± 1.12	129.07 ± 2.31^***&&^

###, *p* < 0.001 vs. WKY. ***, *p* < 0.001 vs. SHR. &&, *p* < 0.01 vs. SB + SJ.

From the results, the combination of SB and SJ can effectively decrease SBP and DBP in SHR, compared with that of SB and SJ single-handed groups. The changes of intestinal flora will influence the antihypertensive effect of the combination of SB and SJ. During the whole experiment process, there was no significant difference in body weight of rats in each group.

### SB and SJ Can Improve Renal Function in SHR

Previous reports have shown that the renal injury indices of SHR were higher than that of WKY rats, including Cr, BUN, and mALB. We measured the relative renal function indices after the treatment of SB and SJ. As shown in Table 3, Cr, BUN, and mALB in SHR were significantly increased compared with WKY(*p* < 0.001, *p* < 0.01, *p* < 0.001, respectively). While SB + SJ significantly decreased the levels of Cr, BUN, and mALB (*p* < 0.01, *p* < 0.05, *p* < 0.01, respectively). Captopril as well as can reduce the production of Cr, BUN, and mALB (*p* < 0.01, *p* < 0.05, *p* < 0.01, respectively). There were no significant functional differences between SB + SJ and captopril. These changes further supported our findings *in vivo* that SB + SJ and captopril treatment attenuate renal injury. Cr, BUN, and mALB in ABS treatment group showed no significant changes compared with SHR group, but Cr, BUN, and mALB in SB + SJ + ABS were significantly lower than those in SHR (*p* < 0.01, *p* < 0.05, *p* < 0.01, respectively), and showed significant changes compared with SB + SJ group (*p* < 0.05, respectively). It indicated that SB and SJ were able to improve kidney function. ABS treatment had no significant effect in comparison with the SHR group, which demostrated that ABS treatment did not affect the normal physiological state of SHR.

**TABLE 3:** Effects of thecombination of SB and SJ on kidney injury based on renal function biomarkers.

Group	WKY	SHR	SB	SJ	SB + SJ	CAP	ABS	SB + SJ + ABS
Cr (μmol/l)	33.18 ± 5.35	73.33 ± 3.34^###^	63.28 ± 4.34	61.38 ± 5.22	49.93 ± 7.05^**^	43.39 ± 4.91^**^	68.04 ± 2.68	59.23 ± 3.01^**&^
BUN (nmol/L)	7.89 ± 0.47	13.18 ± 1.96^##^	10.43 ± 1.56	10.82 ± 0.75	8.46 ± 1.47^*^	8.37 ± 0.36^*^	14.48 ± 1.39	10.79 ± 1.15^&^
mALB (mg/L)	11.70 ± 1.71	64.48 ± 4.89^###^	50.76 ± 4.62	49.65 ± 2.95	42.01 ± 5.15^**^	36.34 ± 5.09^**^	60.55 ± 3.50	49.41 ± 2.71^**&^

###, *p* < 0.001 vs. WKY. ##, *p* < 0.01 vs. WKY. ***,*p* < 0.001 vs. SHR. **, *p* < 0.01 vs. SHR. *, *p* < 0.05 vs. SHR. &&, *p* < 0.01 vs. SB + SJ. &, *p* < 0.05 vs. SB + SJ.

### SB and SJ Can Ameliorate Renal Structure Damage

**FIGURE 2:**
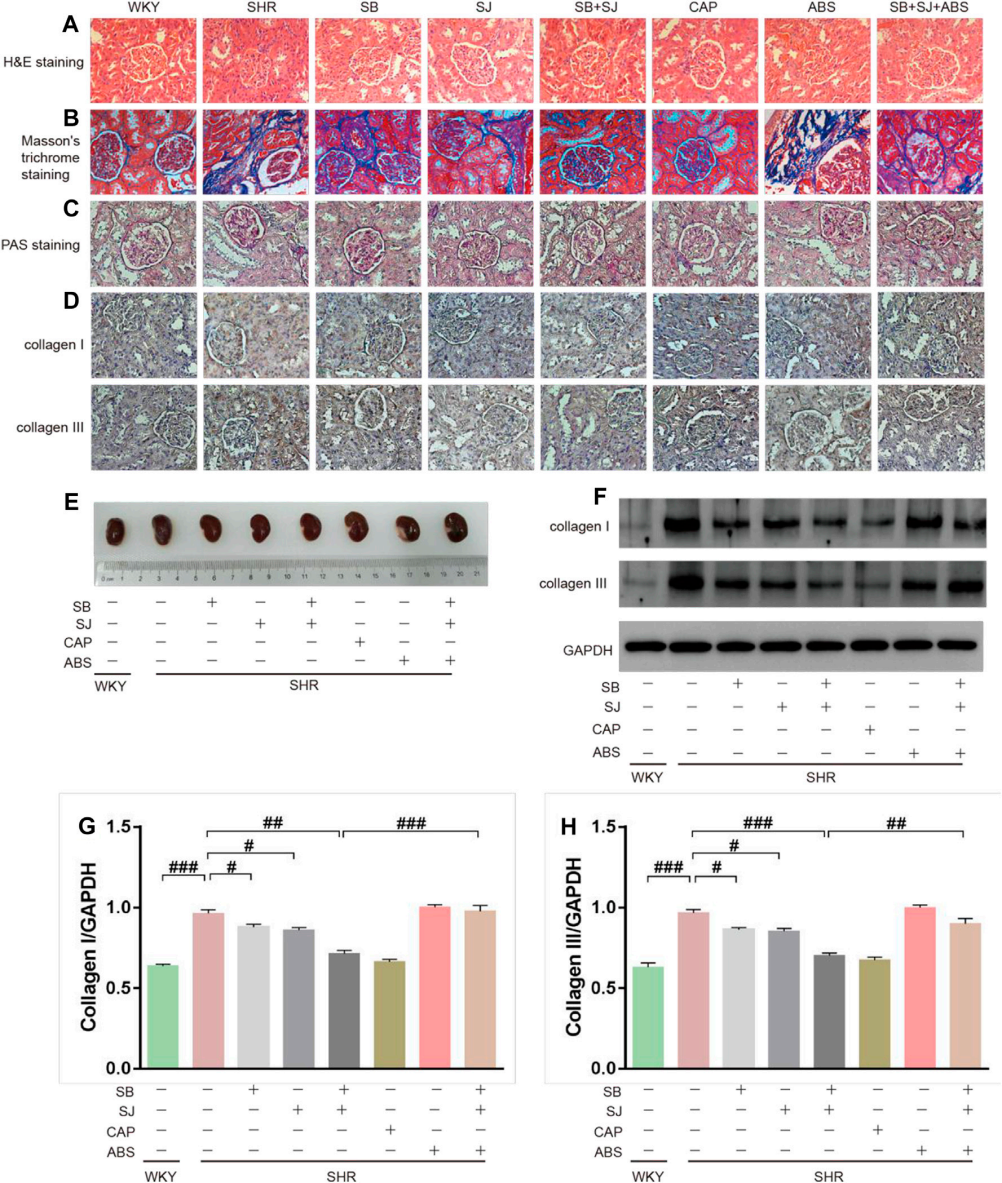
Improvement in renal structure damage by the co-treatment of SB and SJ in SHR. **(A–C)** Renal tissue histology was measured by HE, Masson’s trichrome, and PAS staining. The representative images were taken from the renal tissue slides (Magnification, 40×). **(D)** IHC staining of collagen I or collagen III in the renal tissue; the dark brown staining indicates a positive stain. **(E)** Representative photographs of left kidney tissues. **(F–H)** Western blotting results of collagen I and collagen III and their respective quantification in kidneys. Data are presented as mean ± SD. ###*p* < 0.001. ##*p* < 0.01. #*p* < 0.05.

Upon analyzing the hypertensive effect on kidneys, we found that histological changes in glomeruli and the degree of renal fibrosis (blue collagen staining) in the SB + SJ group were notably alleviated while compared with the SHR group (40× magnification) based on HE and Masson’s trichrome staining. In SHRs, HE staining revealed the proliferation and infiltration of inflammatory cells (Figure 2A), and Masson’s trichrome staining revealed abundant collagen fibers and disruption of the collagen network structure, indicating the formation of kidney fibrosis (Figure 2B). SB + SJ and captopril treatment reduced inflammatory infiltration and protected the renal from fibrotic deposition, inhibited interstitial fibrosis, hypertrophy, and glomerular sclerosis based on PAS staining, in addition, PAS staining indicated that hypertensive renal damage was accompanied with thickening of the basement membranes, (Figure 2C). IHC staining and Western blotting analysis supported that the expression levels of the renal fibrosis marker proteins, collagen I, collagen III were significantly higher in SHRs than those in WKY rats (Figures 2D,E,F, *p* < 0.001, respectively), indicating the formation of kidney fibrosis in SHRs. The expression of collagens in the SB + SJ + ABS group was significantly higher than that in the SB + SJ group (Figures 2G,H), suggesting that depleting the microbiota may affect the renal structure.

### SB and SJ Contribute to Adjust the Disturbance of Intestinal Flora

**FIGURE 3:**
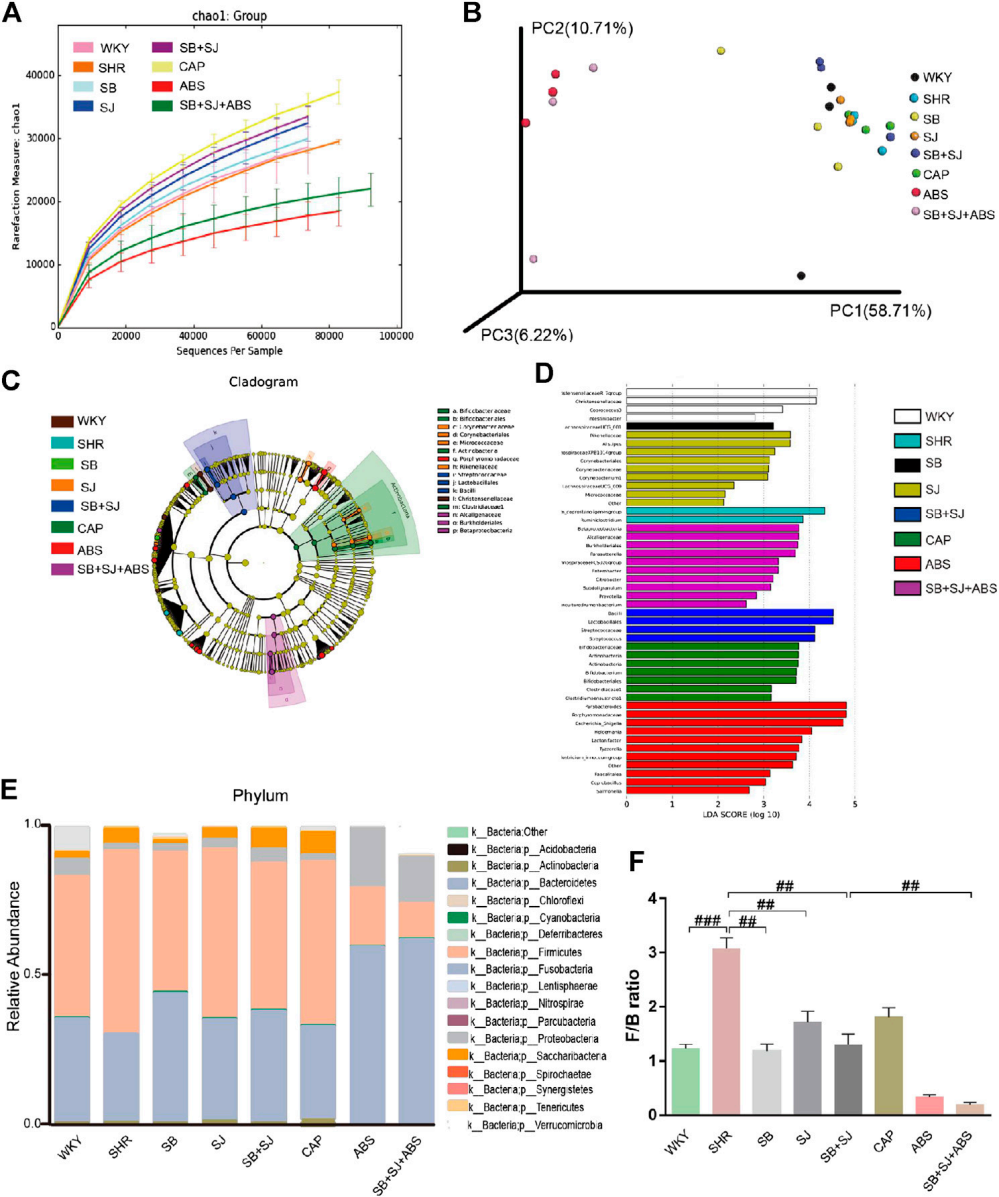
Regulation of gut microbiota by the co-treatment of SB and SJ in SHR. **(A)** The alpha diversity evaluated by PD whole tree. **(B)** Weighted UniFrac-based PCoA analysis of the gut microbiome (beta diversity) via 16S rRNA sequencing. **(C)** Cladogram of LEfSe on the gut microbiome. **(D)** Bar diagram of LEfSe on the gut microbiome. **(E)** The phylum-level composition of the microbiota. **(F)** The Firmicutes/Bacteroidetes ratio. ###*p* < 0.001. ##*p* < 0.01. #*p* < 0.05.

**FIGURE 4:**
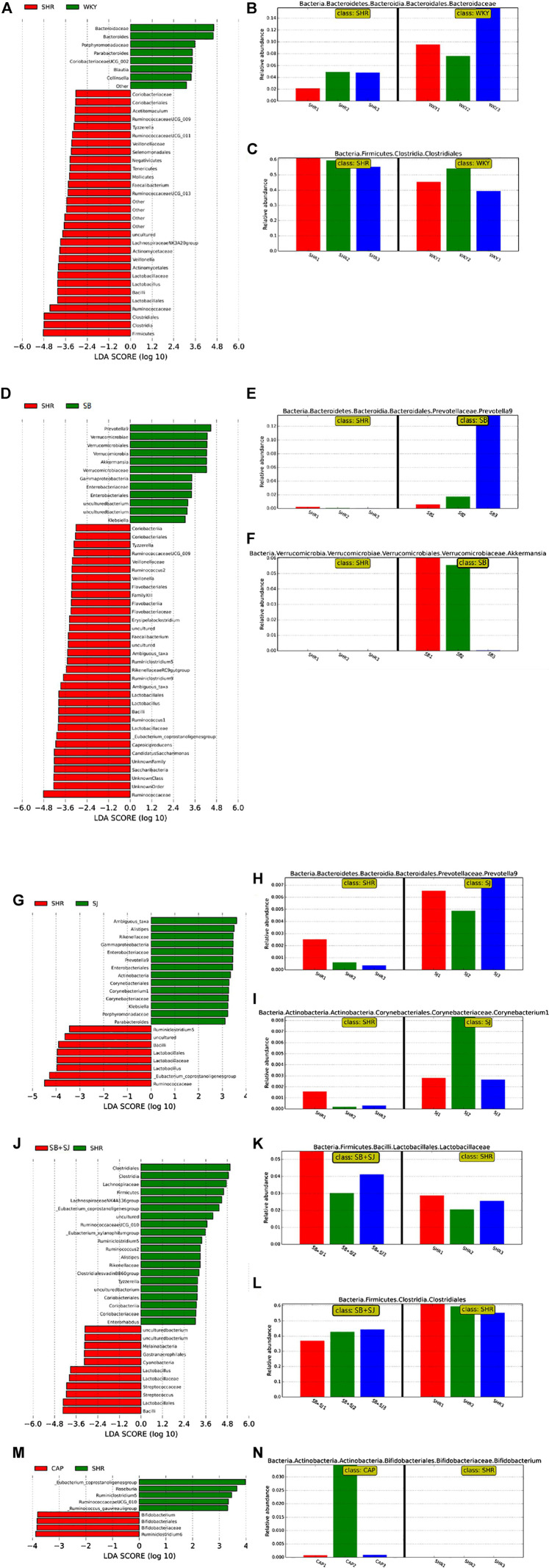
**(A–C)** Bar diagram of LEfSe on the gut microbiome between the WKY and SHR groups. **(D–F)** Bar diagram of LEfSe on the gut microbiome between the SB and SHR groups. **(G–I)** Bar diagram of LEfSe on the gut microbiome between the SJ and SHR groups. **(J–L)** Bar diagram of LEfSe on the gut microbiome between the SB + SJ and SHR groups. **(M,N)** Bar diagram of LEfSe on the gut microbiome between the CAP and SHR groups.

In order to explore whether the treatment of SB and SJ combined with intestinal flora can invoke an even greater effect on inrenal injury, we sequenced the bacterial 16S rRNA V3-V4 region in feces. The analysis of alpha diversity and beta diversity indicated that the variety situation of intestinal flora. As shown in Figure 3A, alpha diversity in the SB + SJ group was higher than in the SHR group, indicating that SB + SJ treatment increased the phylogenetic diversity of gut bacteria. All SHR groups had high beta diversity in the composition of gut microbiota (Figure 3B), which corresponded with LEfSe results (Figures 3C,D) and the different abundances of bacterial phyla (Figure 3E). The phylum-level composition of intestinal flora indicated that the relative abundance of Firmicutes significantly increased and Bacteroidetes decreased in the SHRs compared with the WKY group, while the combination of SB and SJ lowered the ratio of Firmicutes/Bacteroidetes (F/B) at the phylum level in SHRs (*p* < 0.01) (Figures 3E,F). Compared to the WKY group, the relative abundance of Bacteroidaceae was decreased and that of Clostridiales was increased in the SHR group (Figures 4A–C). Compared to SHRs, the relative abundances of Prevotella-9 and Akkermansia were increased in the SB group (Figures 4D–F), while those of Corynebacterium and Prevotella-9 were increased in the SJ group (Figures 4G–I). Meanwhile, the relative abundance of *Lactobacillus* was increased and Clostridiales was decreased in the SB + SJ group (Figures 4J–L). Compared to SHRs, the relative abundance of Bifidobacterium was increased with CAP treatment (Figures 4M,N). Prevotella-9, Lactobacillaceae, and Bifidobacteriaceae are known as beneficial bacteria. Akkermansia, a genus in the phylum Verrucomicrobia, is also known as a beneficial gut microbe, and it has positive relationship with the mucin-producing goblet cells. Clostridiaceae, an indole-positive bacterium, is positively correlated with indole, which has negative effects on kidneys (Warren et al., 2006).

### SB and SJ Contributes to Improve the Integrity of Intestinal Mechanical Barrier

**FIGURE 5:**
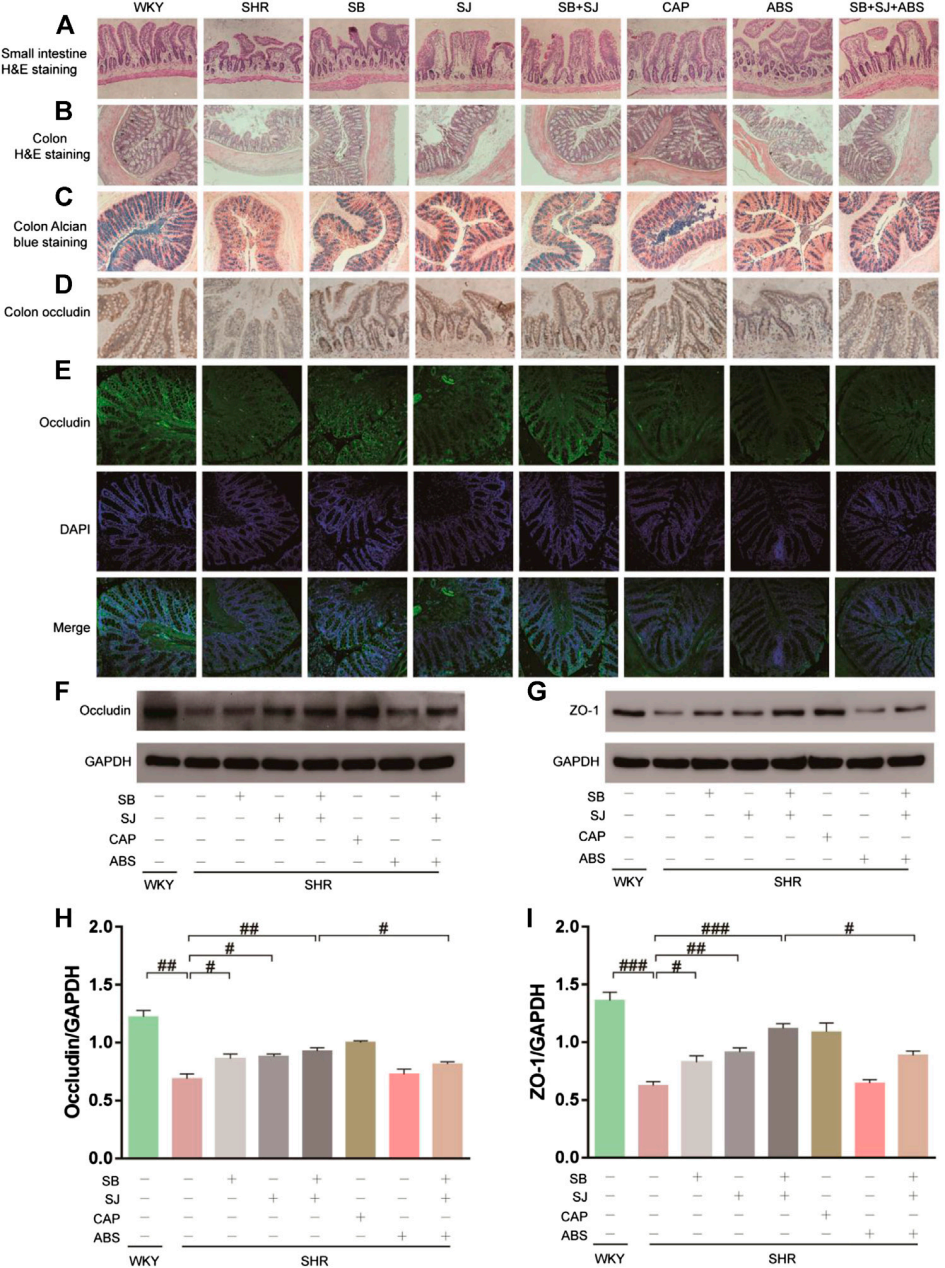
Histological changes showing restoration of the small intestine and colon and gut barrier dysfunction by the combination of SB and SJ in SHR. **(A)** HE staining of the small intestine. The representative images were taken from the gut tissue slides (Magnification, 10×). **(B)** HE staining of colon tissue (Magnification, 10×). **(C)** Alcian blue staining of goblet cells. **(D)** IHC staining of occludin in colon tissue; the dark brown staining indicates a positive stain by occludin. **(E)** The immunofluorescence staining of occludin in colon tissue (Magnification, 20×). **(F–I)** Western blotting results of occludin, ZO-1, and their respective quantification in the colon. Data are presented as mean ± SD. ###*p* < 0.001. ##*p* < 0.01. #*p* < 0.05.

Compared to the WKY group, staining of the small intestine and colon in the SHR group displayed more seriously impaired intestinal structures, including decreases in villus height and colonic goblet cells/crypts, which was partly improved by SB and SJ treatment (Figures 5A–C). One important factor comprising intestinal integrity is tight junction proteins. Therefore, we inspected the expression level of the major tight junction protein in the colon. Higher expression of occludin and ZO-1, which are biomarkers for intestinal barrier integrity, were found in the SB + SJ group compared to that in the SHR group (Figures 5D–G), indicating that hypertension may damage intestinal barrier integrity, and oral administration of SB and SJ may improve the intestinal barrier integrity in SHRs.

### SB and SJ Inhibit the Production of IS and Alleviate Oxidative Stress in Kidneys

**FIGURE 6:**
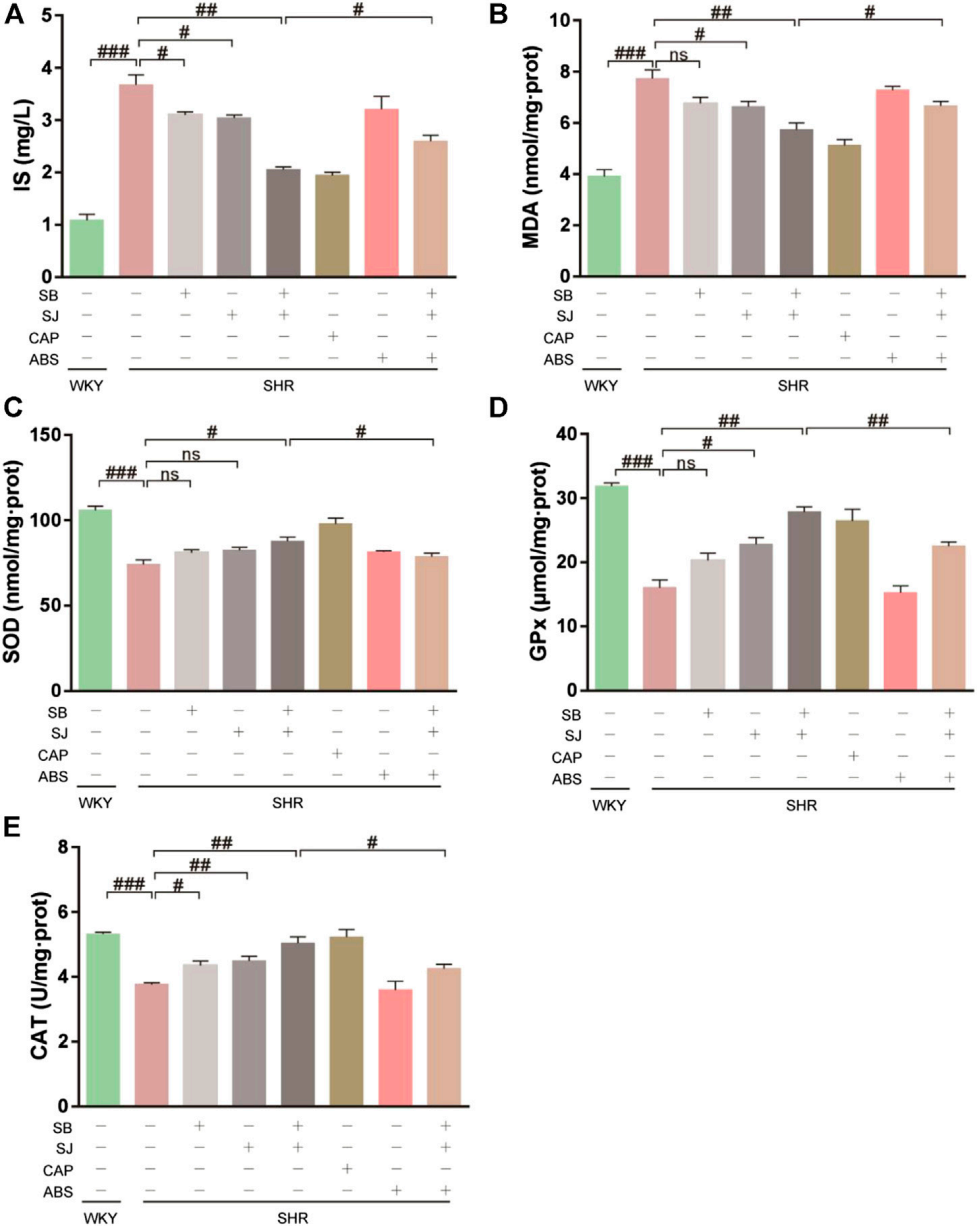
Inhibition of serum IS and oxidative stress in kidneys by the combination of SB and SJ in SHR. **(A)** IS in serum. **(B–E)** The levels of MDA, SOD, GPx, and CAT in kidneys. Data are presented as the mean ± SD. ###*p* < 0.001. ##*p* < 0.01. #*p* < 0.05.

Oxidative stress plays a prominent role in initiating renal fibrosis. IS accumulation occurs early stage of CKD and leads to renal dysfunction by causing fibrosis, inflammation, oxidative stress, and tissue remodeling (Milanesi et al., 2019). During IS production, enteric bacteria metabolize tryptophan from dietary protein into indole, which is absorbed from the portal vein, synthesized in the liver, and then cycled (Mishima et al., 2017). As shown in Figure 6A, SHRs demonstrated higher serum levels of IS and severe oxidative stress in kidneys. This was attenuated by the combination of SB and SJ. As a marker of oxidative stress, the level of renal MDA obviously increased in SHRs compared with that of the WKY group (Figure 6B). The levels of renal SOD, GPx, and CAT, which are considered as a measurement of antioxidant ability, were increased in the WKY group and markedly enhanced in the SB + SJ group (Figures 6C–E). Correspondingly, the effect of combined SB and SJ treatment was reversed by ABS.

### SB and SJ Promote the Production of SCFAs and Regulate the SCFAs Receptors

There is increasing evidence that intestinal microbiota is a key factor in host health and intestinal homeostasis. This is achieved through the release of short chain fatty acids which is the main bacterial metabolites to produce by specific colonic anaerobes after fermentation of dietary fiber and resistant starch, mainly including acetate, propionate, and butyrate. It also reports that SCFAs production was reduced in the course of CKD progression. As shown in Table 4, the levels of acetate, propionate, and butyrate in SHR were significantly decreased compared with that in WKY (*p* < 0.001, respectively). After treatment with SB + SJ, the levels of acetate, propionate, and butyrate were significantly increased (*p* < 0.001, respectively). There was no significant change in SCFAs level between the antibiotic treatment group and SHR group. The level of SCFAs in the SB + SJ + ABS group was significantly higher than that in the SHR group (*p* < 0.01, respectively), and there was a significant difference between the SB + SJ group and the SB + SJ + ABS group (*p* < 0.01, respectively).

**TABLE 4:** Effects of the combination of SB and SJ on fecal SCFAs in SHR.

Group	WKY	SHR	SB	SJ	SB + SJ	CAP	ABS	SB + SJ + ABS
Acetate (μmol/g)	83.65 ± 4.21	62.09 ± 3.87^###^	70.65 ± 1.82^*^	71.18 ± 1.00^*^	80.78 ± 2.91^***^	78.50 ± 3.91^*^	48.76 ± 2.49^*^	73.37 ± 3.24^**&&^
Propionate (μmol/g)	5.23 ± 0.22	3.31 ± 0.17^###^	4.07 ± 0.19^*^	4.08 ± 0.14^**^	4.33 ± 0.18^***^	4.60 ± 0.17^***^	3.36 ± 0.20	3.97 ± 0.21^**&&^
Butyrate (μmol/g)	3.37 ± 0.21	1.44 ± 0.06^###^	2.65 ± 0.21^**^	2.47 ± 0.16^**^	3.10 ± 0.36^***^	2.78 ± 0.16^***^	1.27 ± 0.34	2.01 ± 0.37^&&^

###, *p* < 0.001 vs. WKY. ***, *p* < 0.001 vs. SHR. **, *p* < 0.01 vs. SHR. *, *p* < 0.05 vs. SHR. &&, *p* < 0.01 vs. SB + SJ.

**FIGURE 7:**
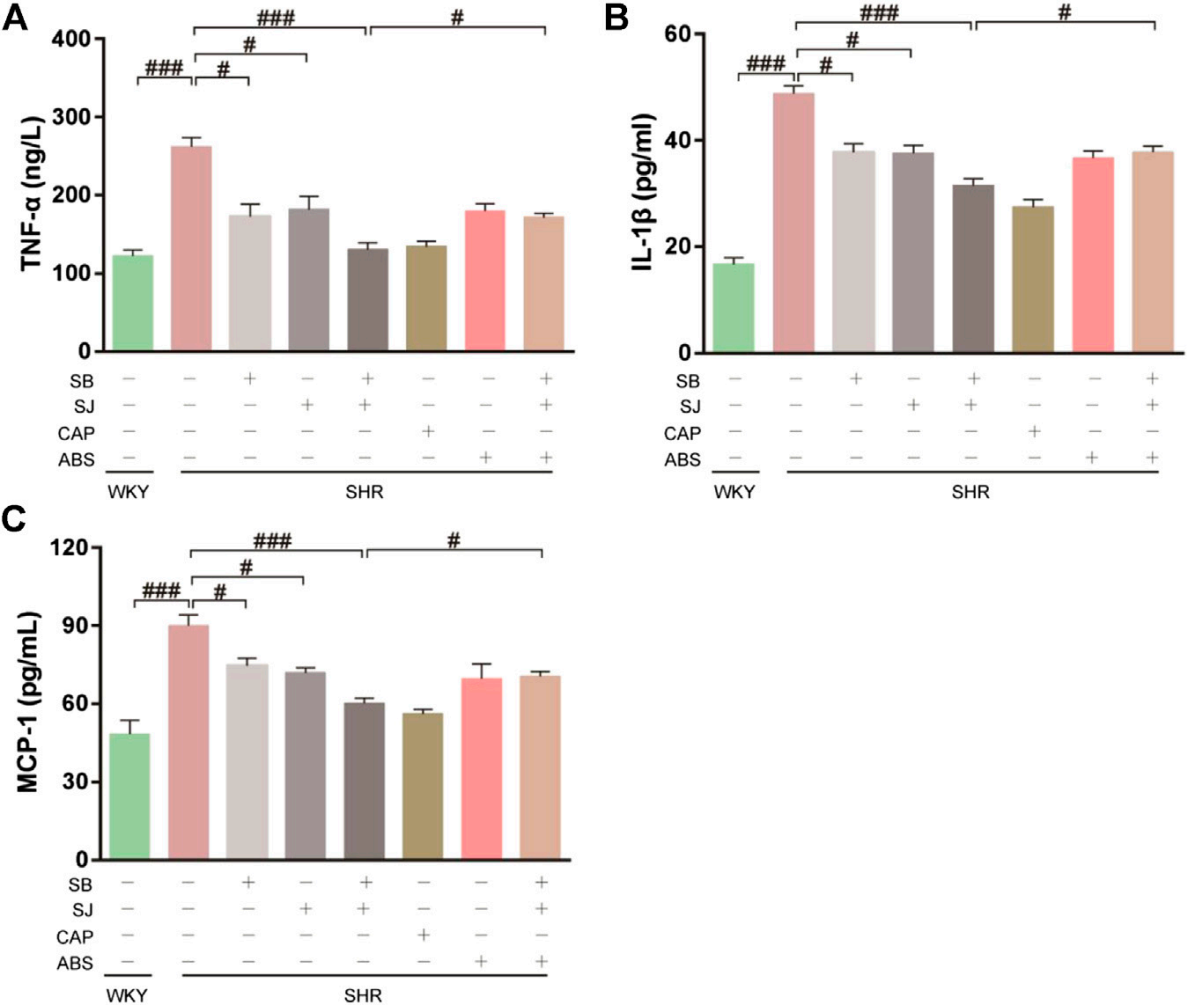
Inhibition of serum inflammation by the combination of SB and SJ in SHR. **(A–C)** Inflammatory markers, including TNF-α, IL-1β, and MCP-1, were assessed. Data are presented as the mean ± SD. ###*p* < 0.001. ##*p* < 0.01. #*p* < 0.05.

Histone deacetylase (HDAC) can deacetylate histone, which can bind to negatively charged DNA and inhibit gene transcription. As ligands of HDAC inhibitors, SCFAs can stimulate monocytes and neutrophils by inducing HDAC inhibition, resulting in the inactivation of NF-κB and reducing the expression of proinflammatory cytokines such as IL-1, MCP-1 and TNF-α. As shown in Figures 7A–C, it was observed the levels of TNF-α, IL-1β, and MCP-1 were higher in SHR serum compared with that in WKY, whereas the levels of inflammatory factors were significantly decreased after the treatment of combination of SB and SJ.

**FIGURE 8:**
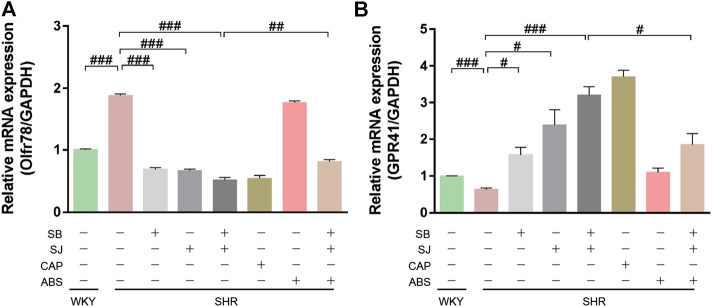
Short-chain fatty acid receptors are altered in the kidneys of SHR. **(A)** The mRNA expression of Olfr78. **(B)** The mRNA expression of GPR41. ###*p* < 0.001. ##*p* < 0.01. #*p* < 0.05.

G protein coupled receptors (GPCRs) are SCFAs receptors, which widely exist in immune cells, intestinal epithelial endocrine cells and other types of cells. Among them, GPR41 can be activated by acetic acid, propionic acid and butyric acid. By activating GPCRs receptors, SCFAs can activate the transductional pathway of immune response and play an anti-inflammatory role in intestinal mucosa. In additional, studies have shown that one of the mechanisms of intestinal microorganisms is to regulate blood pressure through SCFAs. The role of SCFAs in blood pressure regulation involves two receptors: GPR41 and Olfr78. These two receptors showed opposite effects on blood pressure regulation, Olfr78 knockout mice were hypotension while GPR41 knockout mice have hypertension (pluznick, 2017). qPCR analysis results showed that the expression of Olfr78 was increased (Figure 8A) while GPR41 expression was reduced in SHR kidneys (Figure 8B). Compared with SHR group, the treatment of combination of SB and SJ significantly decreased the mRNA expression of Olfr78 (*p* < 0.001) and increased the expression of GPR41 (*p* < 0.001). Olfr78 expression in the SB + SJ + ABS group was significantly higher than that in the SB + SJ group, whereas the opposite was observed with GPR41 expression (Figure 8).

All results suggested that the combination of SB and SJ treatment can increase the expression of SCFAs in SHR, and decrease the inflammatory factors, it also can increase the expression of GPR41 and decrease the expression of Olfr78 as well. These effects can be influenced by the changes of intestinal flora.

### SB and SJ Can Promote the Expression of GLI1

**FIGURE 9:**
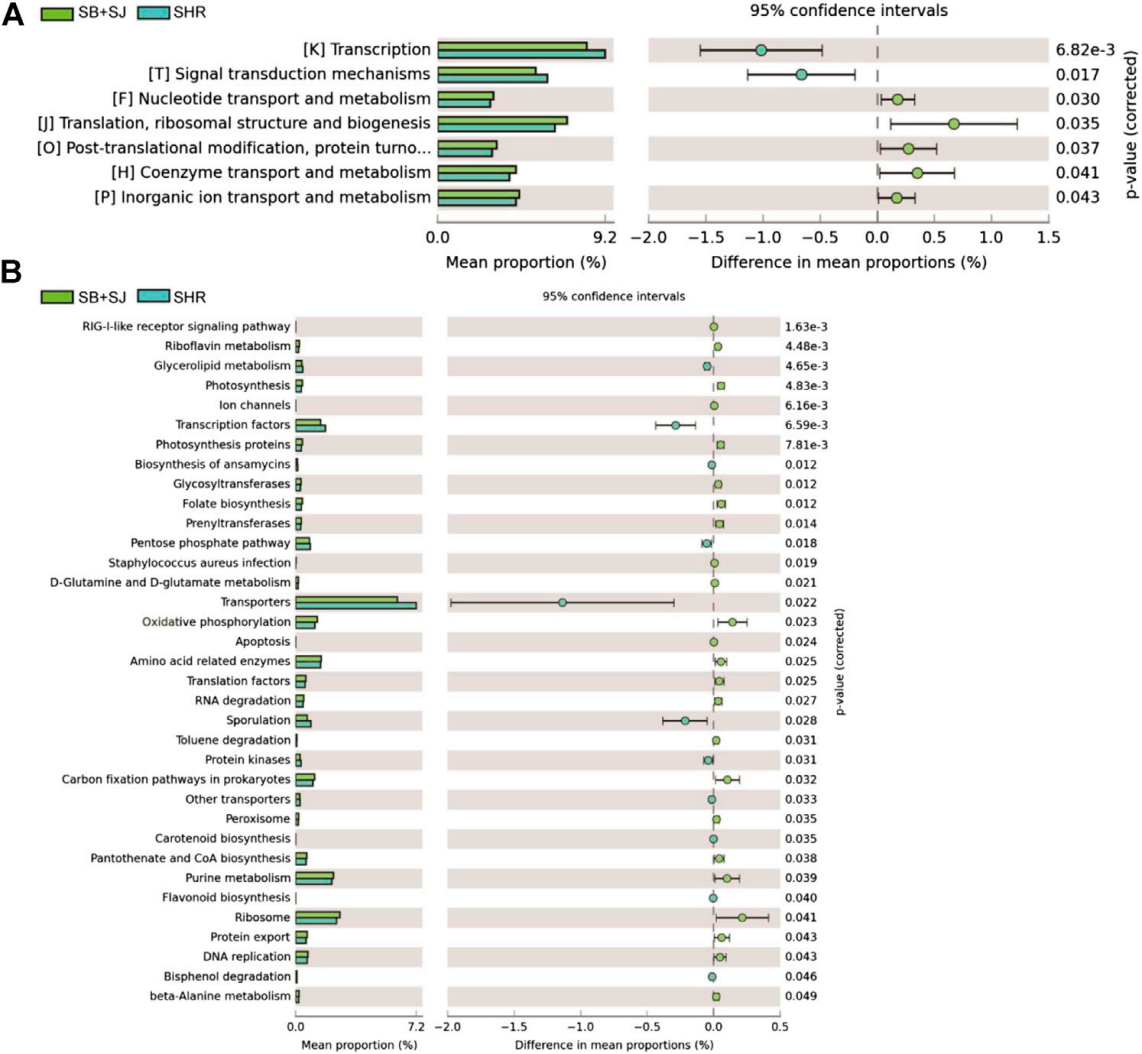
PICRUSt predictions of the functional composition of gut microbiota between the SB + SJ combination group and SHR group. **(A)** COG categories. **(B)** KO categories.

**FIGURE 10:**
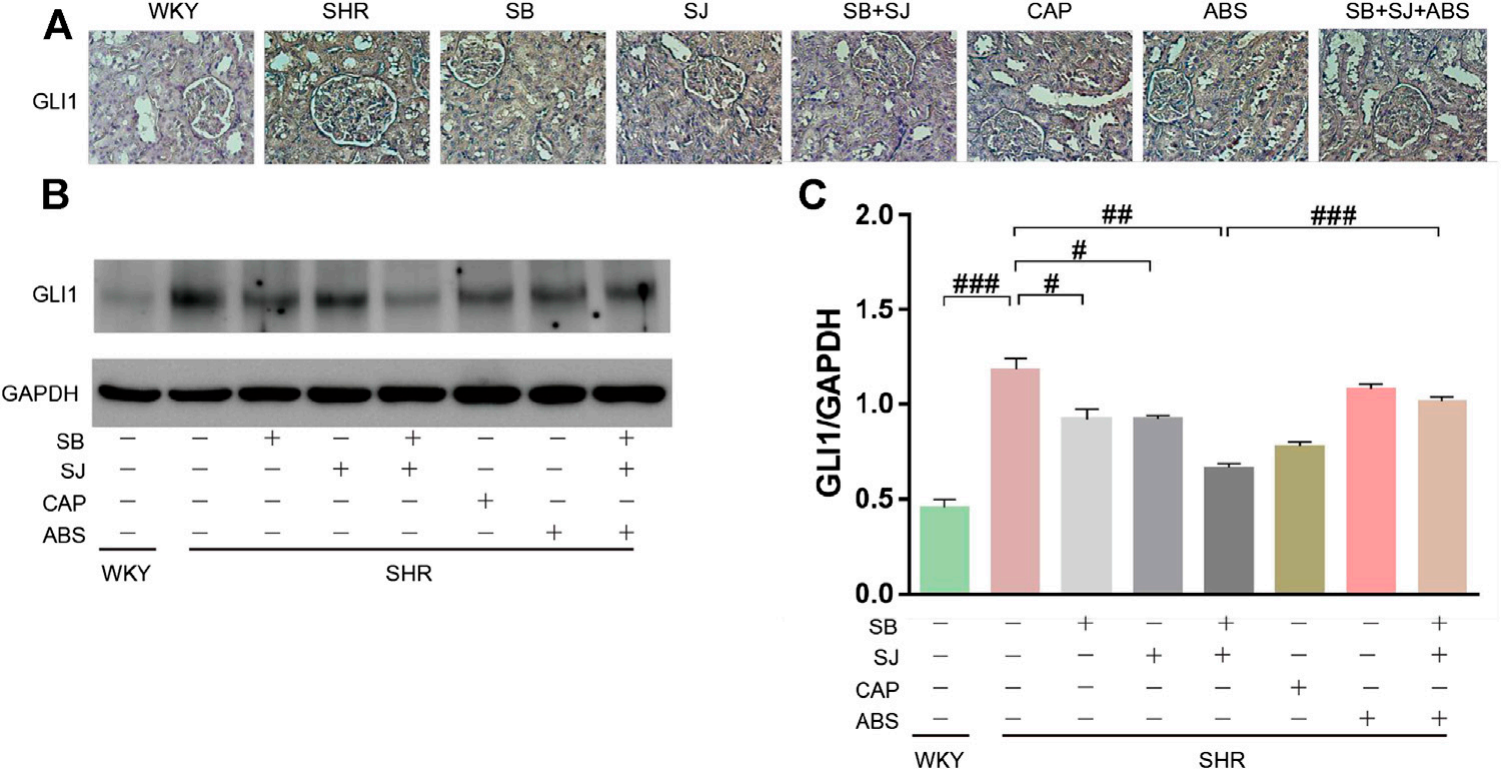
**(A)** IHC staining of GLI1 (Magnification, 40×). **(B,C)** Western blotting results of GLI1 and its quantification in kidneys. Data are presented as mean ± SD. ###*p* < 0.001. ##*p* < 0.01. #*p* < 0.05.

To understand the functional composition of gut microbiota, PICRUSt software (https://picrust.github.io/picrust/index.html) was used to analyze the gene function of putative sample, after determining the species via RNA sequencing, we also analyzed the functional differences among groups using PICRUSt. The results of COG and KO categories showed that the common differential genes closely related to renal fibrosis, hat drawn our attention on the Hedgehog signal pathway (Figures 9A,B). Shh, a kind of protein encoded in the Hedgehog, expresses in the early stage of renal injury, indicating that Hedgehog signal pathway is involved in the process of renal injury. GLI1 is a key transcription factor, which plays an important role in mediating Hedgehog signal pathway (Edeling et al., 2016). Therefore, in the subsequent study, we focused on the Hedgehog signal pathway and transcription factor GLI1. Compared to the SHR group, We observed elevated the expression level of GLI1 in SHR kidney tissues (Figures 10A,B). In addition, SB and SJ co-treatment obviously decreased the expression of GLI1 (*p* < 0.01), while the expression of GLI1 in the SB + SJ + ABS group was markedly higher than that in the SB + SJ group (*p* < 0.001) (Figure 10C). The facts above confirmed that the treatment of SB and SJ can reduce GLI1 expression through regulating gut microbiota.

## Discussion

TCM is regarded as an important and indispensable complementary therapy for hypertensive nephropathy. The combination of SB and SJ that we have devised is a clinical experience prescription, which has been demonstrated to have effect on alleviating kidney injury in hypertensive patients. In our present study, SHRs used as a model of hypertensive nephropathy due to the gradual occurrence of kidney damage with increasing age (Feld et al., 1977; Hultstrom, 2012). As a classic anti-hypertension and kidney-protective drug, captopril, owing to its availability and affordability, has been used extensively in clinical practice; therefore, this drug was selected as a positive control in this work. Our results show that the combination alleviates the histological changes in glomeruli and the degree of renal fibrosis in SHR, suggesting that SB and SJ are conducive to the recovery of renal function and delayed pathological reconstruction.

Recent studies have shown that CKD tends to be accompanied by low-grade inflammation, intestinal flora disorder, and intestinal barrier dysfunction (Vaziri, 2012; Vaziri et al., 2016). Optimal homeostasis in the gut microbiota composition is crucial for protecting the internal homeostasis of the host, while any disturbance of this balance could lead to physiological and pathological conditions (Vaziri et al., 2016; Shen, 2017), and 12 healthy persons revealed that there were obvious distinctions in the abundance of 190 bacteria between end-stage renal disease (ESRD) and healthy controls. An imbalance in gut microbiota is quantified the changes via the ratio of F/B, which can be used as a biomarker for pathological conditions (Mariat et al., 2009; Hevia et al., 2014; Koliada et al., 2017). Here, the ratio of F/B in the colonic digest of the SB + SJ group was remarkably decreased compared to that in the SHR group. In addition, in order to clarify whether SB and SJ produced effects through the intestinal flora pathway, thus, we designed rescue experiments by establishing an antibiotic control group and antibiotic + SB + SJ combination group. ABS treatment was used to deplete intestinal microflora and simulate a germ-free spontaneous hypertensive nephropathy model.

LEfSe was employed to analyze the specific phylotypes. This analysis revealed that Prevotella-9 and Akkermansia were increased with SB treatment, while the relative abundance of Corynebacterium and Prevotella-9 were increased with SJ treatment. *Lactobacillus* was markedly increased and Clostridiaceae was decreased with co-administration of SB and SJ after 15 weeks, while Bifidobacterium was increased with captopril treatment. Among these microbiota, *Lactobacillus* and Akkermansia were associated with intestinal permeability and tight junction expression (Cani and de Vos, 2017; Robles-Vera et al., 2018), Clostridiaceae (Warren et al., 2006) and Bifidobacteriaceae (Kimura et al., 1995; Niwa, 2013) were linked to the indoxyl metabolic pathway. Next, we will discuss the role of dominant microflora in reversing kidney injury.

It is generally believed that the intestinal mucosal barrier is comprised of mechanical, chemical, and immune barriers. The intestinal mucosal barrier can prevent harmful microorganisms and toxic metabolites from entering the blood; however, the mucosal barrier is damaged in patients with CKD. Recent research has found that hypertension increases the permeability of the intestinal epithelial barrier (Santisteban et al., 2017; Kim et al., 2018), which is associated with inflammation and the pathological progression of many diseases, such as obesity, diabetes, and other metabolic disorders. Our study demonstrated that compared to the WKY rats, the intestinal barrier of SHRs was characterized by structural damage of colonic mucosa, mucous deficiency, and exfoliation of epithelial cells on the surface of the mucosa group. Through subsequent the gut microbiota analysis, we found that the SB + SJ co-treatment group could increase *Lactobacillus*, which is identified to participate in repairing the intestinal mucosal barrier. Robles-Vera et al. (Robles-Vera et al., 2018) found that *Lactobacillus* treatment obviously enhanced intestinal integrity and may prevent harmful bacteria and their antigens from passing through the intestinal epithelium. After treatment with SB + SJ and captopril, the mucosal damage in the intestinal barrier was repaired. Meanwhile, tight junction proteins, including occludin and ZO-1, were overexpressed. The results suggested that the mechanism by which the combination of SB + SJ aids in repairing the intestinal barrier might be associated with increasing the content of intestinal *Lactobacillus*.

The gut-renal axis theory holds that the kidneys and intestines influence each other. Intestinal flora disorders lead to changes in metabolites such as indole, trimethylamine N-oxide, and SCFAs. Increased intestinal permeability raises metabolite levels in the blood, thus accelerating the progression of kidney injury and complications (Mahmoodpoor et al., 2017).

IS is a metabolite from Enterobacteriaceae (Wikoff et al., 2009). IS can accumulate continuously in CKD and combine with proteins, damage renal tubular epithelial cells, and lead to renal interstitial fibrosis (Milanesi et al., 2019). Gut microbiota analysis showed that Bifidobacteriaceae, an indole-negative bacterium, was decreased, whereas Clostridiaceae, an indole-positive bacterium, was increased in SHRs. The serum level of IS was significantly increased in SHRs compared with WKY rats. Meanwhile, the oxidative stress pathway was activated by IS, related oxides such as MDA were increased, and SOD, GPx, and CAT were remarkably decreased. After intervention with SB + SJ and captopril, the above mentioned changes were reversed: Bifidobacteriaceae was increased, Clostridiaceae was decreased, IS serum levels were significantly decreased, and the oxidative stress pathway was inhibited. Meanwhile, the SB + SJ + antibiotics group and SB + SJ group showed a significant difference.

The probiotic metabolite SCFAs in the intestine of patients with CKD are reduced, and the decrease in SCFAs can activate systemic inflammation. In addition to bacterial metabolites, endotoxins from intestinal bacteria, such as lipopolysaccharide (LPS), can stimulate immune cells, especially macrophages and endothelial cells, to activate innate immune responses and secrete various pro-inflammatory cytokines (e.g., TNF-α and IL-1β) by forming a complex with Toll-like receptors, eventually leading to systemic inflammatory response and aggravating oxidative stress (Marchesi et al., 2016). We found high levels of pro-inflammatory cytokines in SHR serum, while after treatment with SB and SJ, these levels were reduced significantly. Rescue experiments revealed that the serum levels of TNF-α, IL-1β, and MCP-1 in the SB + SJ + antibiotic group were remarkably higher than those in the SB + SJ group, suggesting that the combination of SB and SJ decreased inflammatory cytokines by regulating gut microbial ecology.

Some research has confirmed that SCFAs produced by intestinal flora may regulate blood pressure by acting on two key SCFAs receptors, Olfr78 and GPR41 (Pluznick, 2014). Olfr78 is expressed in kidneys and regulates renin secretion, which is able to increase blood pressure (Pluznick et al., 2013), while GPR41 is involved in lowering blood pressure (Natarajan et al., 2016). In this study, we found that compared with the results of WKY rats, fecal SCFAs such as acetate, propionate, and butyrate were decreased in SHRs; correspondingly, the expression of Olfr78 mRNA was increased while that of GPR41 was decreased in SHRs. This change may be associated with improving vasoconstriction, leading to renal damage and a continuous hypertensive condition. With SB and SJ co-treatment, the mRNA expression of Olfr78 decreased while that of GPR41 remarkably increased compared with the untreated SHR group. GPR41 expression was significantly lower after SB and SJ co-treatment under the germ-free conditions. This indicated that SB and SJ can reverse renal injury through modulating blood pressure regulator receptors by increasing the levels of SCFAs, and this function is dependent on gut microbiota.

Moreover, accompanied by PICRUSt analysis, and COG and KO category predictions, we found clearly enhanced transcription factors in the SB + SJ group. GLI1 has aroused our interest among transcription factors due to its key role in mediating Hh signal pathway, which has a vital functional role in fibrosis development in kidneys (Edeling et al., 2016). IHC and Western blot results showed that GLI1 expression was increased in SHRs compared with those in WKY rats, and SB and SJ co-treatment reduced GLI1 expression significantly. Similarly, GLI1 expression in the SB + SJ + antibiotics group was notably higher than that in the SB + SJ group. It was confirmed that SB and SJ reduced GLI1 expression through regulating gut microbiota.

## Conclusion

Our study demonstrated that oral administration of SB and SJ could lower blood pressure and reverse renal injury in SHRs. The mechanism is involved in the correction of gut microbiota and SCFAs or metabolites. Our study suggests that the rebuilding of the gut microbiota may be helpful to protect the intestinal mucosal barrier. The increase in dominant bacteria can reduce IS accumulation and further inhibit the activation of the oxidative stress pathway. In addition, the combination of SB and SJ can increase the content of SCFAs, which are able to inhibit the release of inflammatory factors and reduce blood pressure by decreasing the expression of Olfr78 and increasing that of GPR41 to alleviate kidney damage. The aforementioned actions are realized through regulation of the gut microbiota. However, our study has some limitations, for instance, although we have found the specific gut microbiota in a different group, the mechanism by which some flora affects kidney damage remains unclear and requires further investigation.

## Data Availability

The original contributions presented in the study are publicly available. This data can be found here: https://www.ncbi.nlm.nih.gov/ PRJNA679453.

## Glossary

ABS: Antibiotics
AJ: adhesion junction
BSA: bovine serum albumin
BUN: blood urea nitrogen
CAT: catalase
CAP: Captopril
CE: collision energy
CES: collision energy expansion
CKD: chronic kidney disease
COG: Clusters of Orthologous Groups
Cr: creatinine
Ct: comparative threshold cycle
DBP: diastolic blood pressure
DBS: dynamic background subtraction;
ESRD: end-stage renal disease
GJ: gap junction
GPCRs: G protein coupled receptors
GPR41: G protein couple receptor 41
GPx: glutathione peroxidase
HDAC: Histone deacetylase
IDA: information dependent acquisition
IHC: Immunohistochemistry
IL-1: interleukin-1
IS: Indoxyl sulfate
KO: KEGG Orthology
LefSe: Linear discriminate analysis effect size
LPS: lipopolysaccharide
mALB: microalbumin
MDA: malondialdehyde
MCP-1: monocyte chemotactic protein-1
NF-κB: nuclear factor kappa-B
Olfr78: olfactory receptor 78
OTUs: operational taxonomic units
PcoA: principal coordinate analysis
PICRUSt: Phylogenetic investigation of communities by reconstruction of unobserved states
SBP: systolic blood pressure
SCFAs: short chain fatty acids
SHR: spontaneously hypertensive rats
SB: Scutellaria baicalensis Georgi
SD: standard deviation
SJ: *Sophora* japonica L
SOD: superoxide dismutase
TCM: traditional Chinese medicine
TJ: tight junction
TNF–α: tumor necrosis factor–α
WKY: Wistar-Kyoto
