# Supplementary material for: The Combination of Scutellaria baicalensis Georgi and Sophora japonica L. ameliorate Renal Function by Regulating Gut Microbiota in Spontaneously Hypertensive Rats
Source: Front Pharmacol. 2021 Feb 12;11:575294. doi: 10.3389/fphar.2020.575294 (PMC7907655; doi:10.3389/fphar.2020.575294)

Supplement Figure 1. Mass spectra of the active ingredients in *Scutellaria baicalensis Georgi* and *Sophora japonica L.*

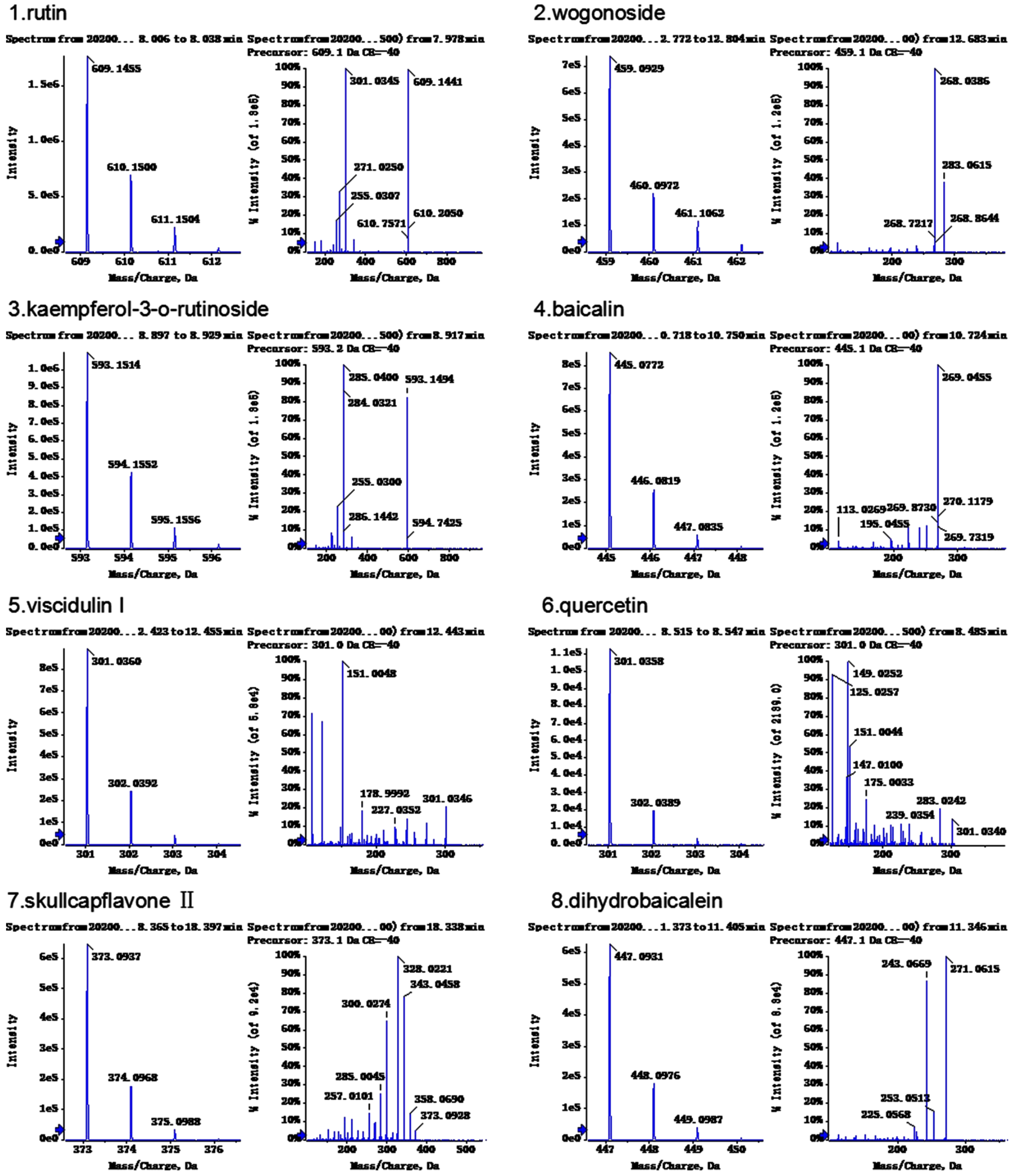

9.dihydrobaicalin

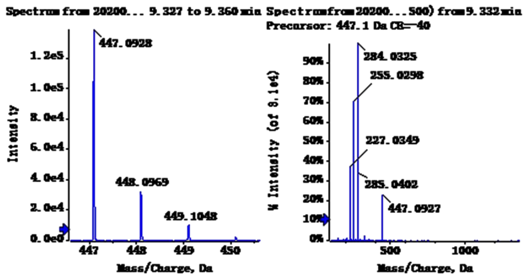

10.baicalein

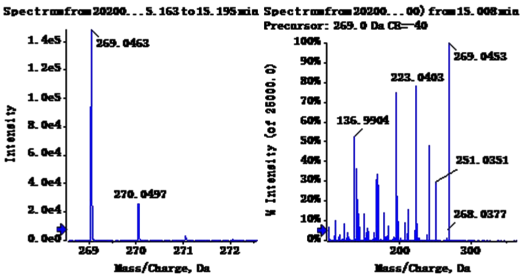

11.norwogonin

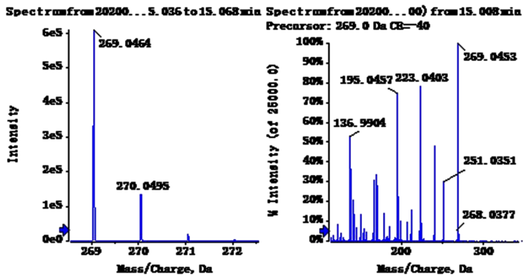

12.genistein

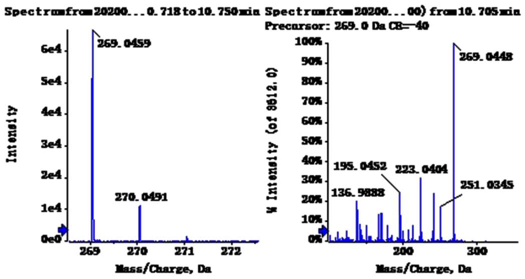

13.kaempferol

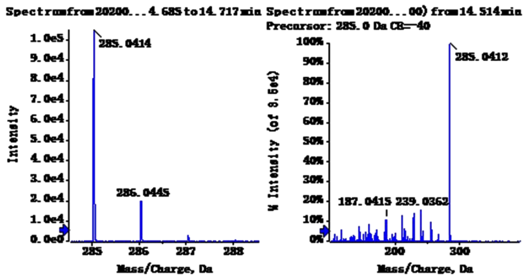

14.isorhamnetin

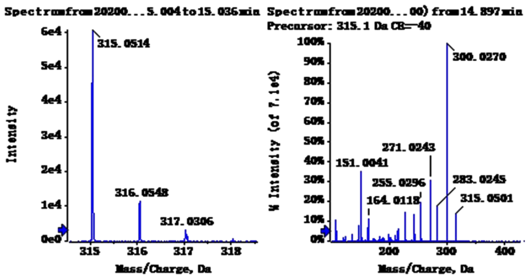

15.wogonin

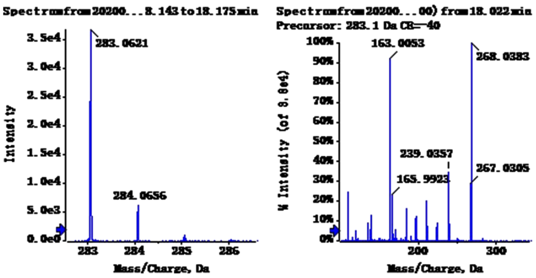

16.viscidulin III

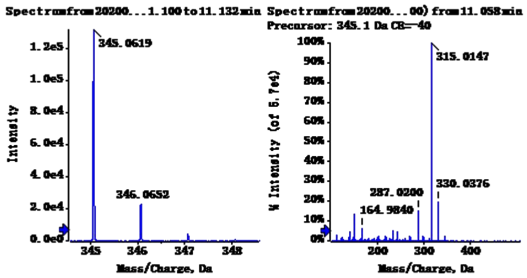

25.skullcapflavone I

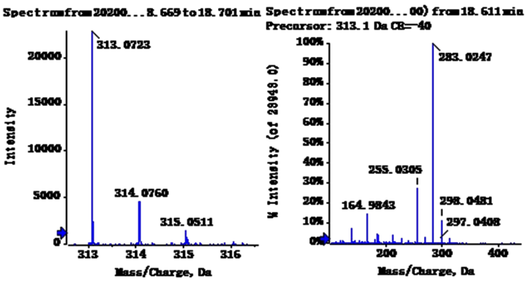

26.viscidulin II

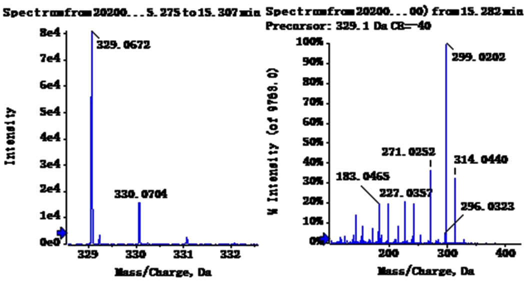

27.7,2'6'-trihydroxy-5-methoxydihydroflavone

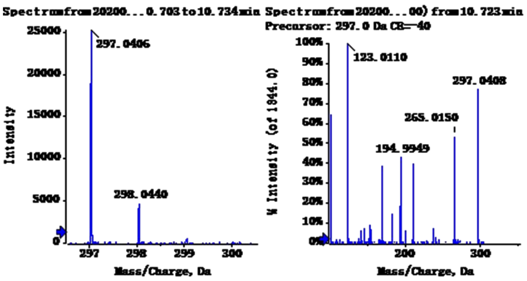

28.palmitic acid

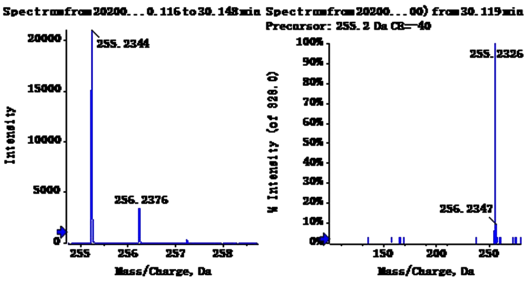

29.dihydrooroxylin A

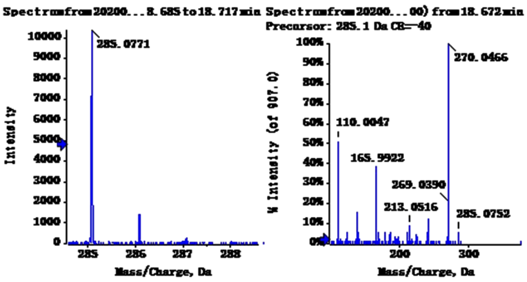

30.2',6',5,7-tetrahydroxyflavan-one

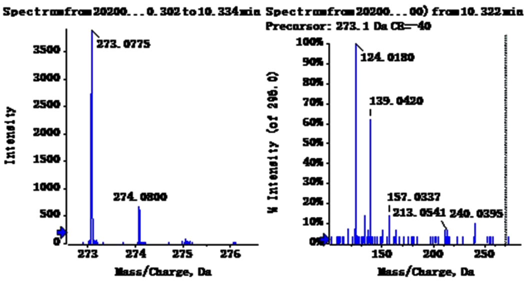

31.stearic acid

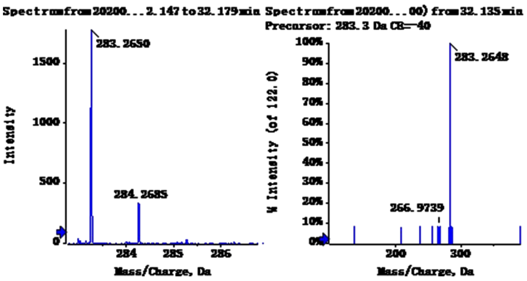

17.chrysin

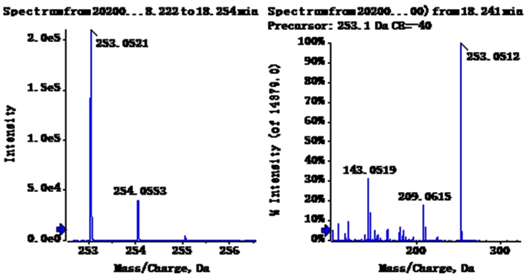

18.soyasaponin Bb

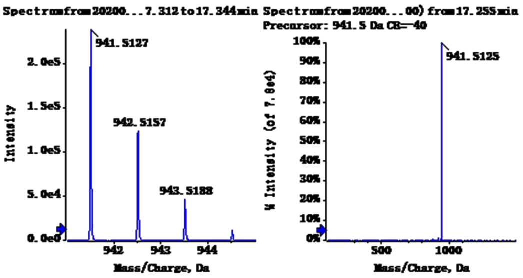

19.sophora saponin III

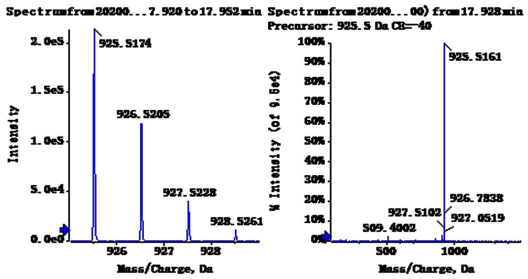

20.2',5,6',7-tetrahydroxyflavanonol

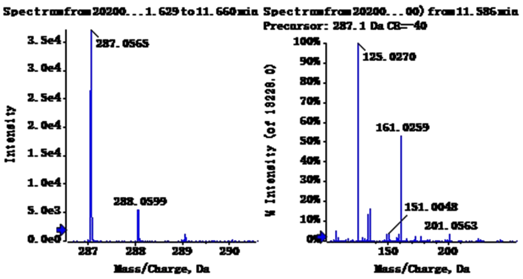

21.eriodictyol

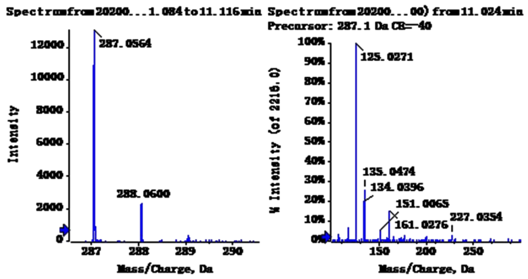

22.2,6,2',4'-tetrahydroxy-6'-methoxychalcone

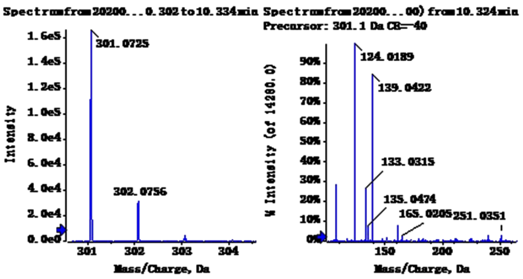

23.dianbaicalin

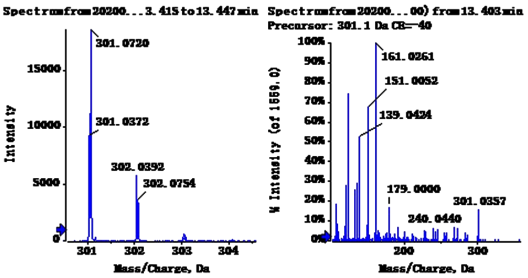

24.skullcapflavon

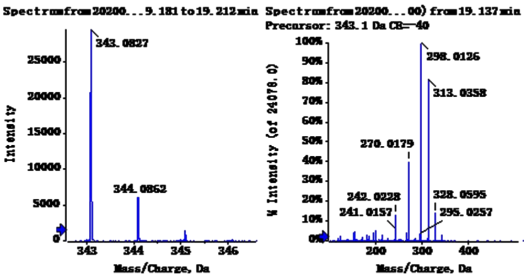

Supplement: Supplementary file 1 [file image1.pdf]
